# Adsorption-Governed Plasmonic Sensing for Environmental Management and Monitoring Programs

**DOI:** 10.3390/mi17101168

**Published:** 2026-10-07

**Authors:** Yuan-Fong Chou Chau, Abdul Hanif Mahadi, Roshan Thotagamuge, Muhammad Raziq Rahimi Kooh

**Affiliations:** 1 Centre for Advanced Material and Energy Sciences, Universiti Brunei Darussalam, Tungku Link, Gadong BE1410, Brunei; hanif.mahadi@ubd.edu.bn; 2 Applied Physics, Faculty of Science, Universiti Brunei Darussalam, Tungku Link, Gadong BE1410, Brunei; 3 Chemical Sciences, Faculty of Science, Universiti Brunei Darussalam, Tungku Link, Gadong BE1410, Brunei; 4 Department of Optometry, Faculty of Allied Health Sciences, University of Sri Jayewardenepura, Nugegoda 10250, Sri Lanka; roshan@sjp.ac.lk

**Keywords:** plasmonic adsorption, SPR, LSPR, SERS, environmental monitoring

## Abstract

Plasmonic environmental sensors are often characterized by optical enhancement, but their field performance is fundamentally governed by adsorption at the sensing interface. This review examines adsorption-controlled plasmonic sensing across water, air, soil, and food-environment matrices, emphasizing environmental management rather than cross-domain plasmonic applications. This review evaluates surface plasmon resonance, localized surface plasmon resonance, and surface-enhanced Raman scattering in terms of pretreatment requirements, matrix interference, fouling, regeneration, reproducibility, validation against standard methods, and technology readiness. The review shows that plasmonic platforms are most valuable when used as rapid screening, distributed, or early-warning tools that complement, rather than replace, reference methods such as ICP-MS, GC-MS, and LC-MS/MS. Practical translation requires matrix-matched calibration, antifouling strategies, stronger reporting standards, and side-by-side comparison with established analytical workflows. This paper proposes a management-oriented framework for deploying plasmonic sensors in tiered monitoring programs and integrating results into regulatory, operational, and decision-support contexts.

## 1. Introduction

Plasmonic adsorption-enabled sensing translates nanoscale light–matter interactions into chemical and environmental analysis by coupling optical transduction with interfacial adsorption. In surface plasmon resonance (SPR), localized surface plasmon resonance (LSPR), and surface-enhanced Raman scattering (SERS) platforms, the sensing response depends not only on plasmonic excitation but also on how target molecules adsorb, orient, accumulate, and chemically interact at the plasmonic interface. Adsorption changes the local dielectric environment, modifies near-field coupling, and can induce charge-transfer effects that alter Raman polarizability and plasmon damping [1,2,3,4,5,6,7]. Thus, adsorption should be treated as a central design variable rather than a secondary surface event.

For environmental sensing, high optical sensitivity alone is insufficient. Real samples contain natural organic matter, salts, surfactants, biofilms, and competing ions that can occupy active sites, shift baselines, suppress target binding, or generate false signals [8,9,10,11,12,13,14]. Reliable detection therefore requires selective adsorption, antifouling interfaces, stable surface chemistry, and reproducible signal generation across water, air, soil, and food-related matrices. In many plasmonic environmental sensors, the main performance bottleneck is not the optical transducer but the adsorption layer that connects the sample to the optical readout.

**Figure 1:**
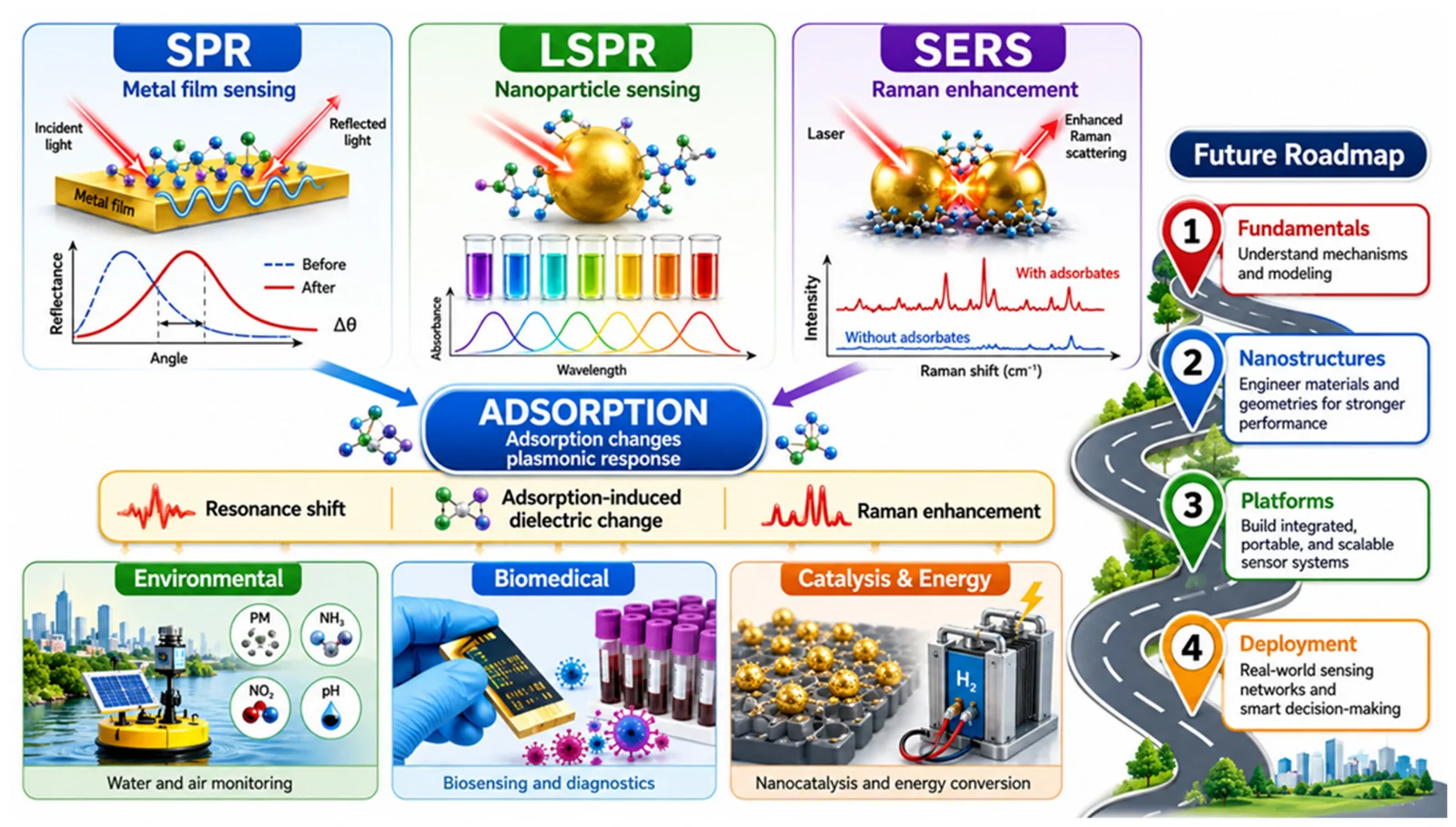
Adsorption-driven plasmonic sensing: adsorbates on SPR, LSPR, or SERS platforms induce dielectric changes, yielding resonance shifts or Raman enhancement. Applications span environmental, biomedical, and energy sectors; A four-step roadmap links fundamental adsorption to field-deployable intelligent systems.

Figure 1 shows adsorption as the key link between plasmonic nanostructures and sensing outputs: adsorbed molecules alter optical properties, producing SPR/LSPR shifts or SERS enhancement. This principle applies to environmental monitoring, biomedical sensing, catalysis, and energy conversion.

The three optical responses in Figure 1 originate from different interactions with the same adsorption interface. In film-based SPR, a bound layer changes the effective dielectric environment sampled by a propagating evanescent mode, shifting the reflectance minimum; its magnitude depends on surface coverage and the layer’s refractive index and thickness. An LSPR nanoparticle probes a shorter, geometry-dependent near-field, so molecules bound near tips or gaps can contribute more than equally numerous molecules farther away. SERS instead requires analytes to occupy accessible high-field sites and provides vibrational features rather than a refractive-index-only signal. Interfacial charge transfer may alter molecular polarizability for a suitably coupled adsorbate, but enhancement alone should not be used to infer it. The roadmap therefore begins with adsorption chemistry and field overlap, then progresses to matrix-tolerant interfaces, calibrated readout, and validated deployment.

Supplementary Table S1 summarizes representative plasmonic nanostructures, including colloids, nanorods, core–shell systems, nanoporous substrates, ordered arrays, and hybrid composites with polymers, metal–organic frameworks (MOFs), semiconductors, and 2D materials. These examples highlight morphology, surface area, hot-spot density, and interfacial chemistry as key tunable parameters.

Environmental monitoring is a particularly important testbed for adsorption-controlled plasmonic sensing. Conventional techniques such as chromatography and mass spectrometry remain essential for high-accuracy analysis, but they are often slow, costly, and centralized [11,12]. Plasmonic sensors offer portable, low-volume, and real-time detection, but their field value depends on reliable operation in untreated or minimally treated samples. Supplementary Figure S1 shows adsorption as the key step in plasmonic environmental sensing: after sampling and optional pretreatment, analytes must selectively bind to functionalized recognition layers before SPR, LSPR, or SERS transduction. Matrix interferences such as NOM, salts, and biofouling can disrupt adsorption, making real-world performance limited more by interfacial binding efficiency than by optical sensitivity.

Next-generation plasmonic environmental sensors should therefore be designed as adsorption-aware systems rather than isolated optical devices. Key requirements include antifouling surfaces, selective receptors, regenerative interfaces, stable nanostructures, portable optics, and data-driven calibration. Machine learning (ML) can support this transition by distinguishing target-induced signals from drift, broadening, nonspecific adsorption, and matrix noise. Sustainable materials, scalable fabrication, disposable cartridges, and standardized testing protocols will also be critical for commercialization and field deployment.

This review frames plasmonic sensing as an integrated adsorption–transduction process involving nanostructure design, surface chemistry, adsorption thermodynamics and kinetics, and optical response. SPR and LSPR rely on adsorption-induced resonance shifts in angle or wavelength, whereas SERS requires analyte localization within electromagnetic hot spots and, in favorable cases, charge-transfer-assisted enhancement. Across these modalities, interfacial adsorption behavior governs signal-to-noise ratio (SNR), selectivity, response time, reversibility, and long-term stability. This review identifies adsorption as the key interfacial factor connecting plasmonic transduction with selectivity, matrix tolerance, regeneration, and field readiness. To define its contribution, we compare recent reviews across four dimensions: mechanistic coverage of interfacial binding, treatment of environmental matrices, depth of performance benchmarking, and emphasis on field translation.

This structured narrative review focuses on environmental monitoring and draws on literature from Web of Science, Scopus, and major publisher databases. Search terms included plasmonic sensing, SPR/LSPR/SERS, surface functionalization, fouling, molecularly imprinted polymers, and field-deployable sensors. We prioritized studies published from 2018 onward, while including earlier foundational work where needed to explain core mechanisms. We selected articles based on relevance to adsorption-mediated optical responses, validation in realistic matrices, and translational significance. We considered biomedical, catalytic, and photonic examples only when their interfacial principles were directly transferable to environmental sensing.

The review moves from fundamental mechanisms to practical deployment. It first explains how interfacial binding perturbs plasmonic modes, drives SPR/LSPR shifts, and enables SERS enhancement. It then compares relevant nanostructures, surface chemistries, hybrid materials, and recognition layers. It assesses applications in water, air, soil, and food for matrix interference and field applicability, and discusses selected broader examples only to provide transferable design insights. The review concludes by outlining priorities in interface engineering, AI-assisted spectral analysis, scalable fabrication, sustainability, standardization, and commercialization of intelligent plasmonic sensing platforms.

## 2. Fundamentals of Plasmonic Adsorption Mechanisms

### 2.1. Surface Plasmon Resonance (SPR): Propagating Modes and Adsorption-Driven Resonance Shifts

SPR converts adsorption at a metal–dielectric interface into a measurable optical shift. When target molecules bind to a functionalized metal film, they form an interfacial layer with an effective refractive index and thickness that differ from those of the surrounding medium. This modifies the resonance condition and changes the resonance angle or wavelength, allowing real-time tracking of association, equilibrium, and dissociation processes [15,16,17].

The main conclusion is that SPR sensitivity is fundamentally governed by the quality of the adsorption layer rather than by optical interrogation alone. The evanescent field probes only the near-surface region, so surface coverage, molecular conformation, binding strength, and fluctuations in the background refractive index directly affect the signal. Because SPR measures the effective optical thickness rather than chemical identity, similar resonance shifts may arise from different adsorbates, nonspecific binding, temperature drift, or matrix-induced changes. Therefore, reliable SPR environmental sensing requires selective recognition coatings, reference channels, antifouling surfaces, and calibration strategies that separate target adsorption from background variation.

**Figure 2:**
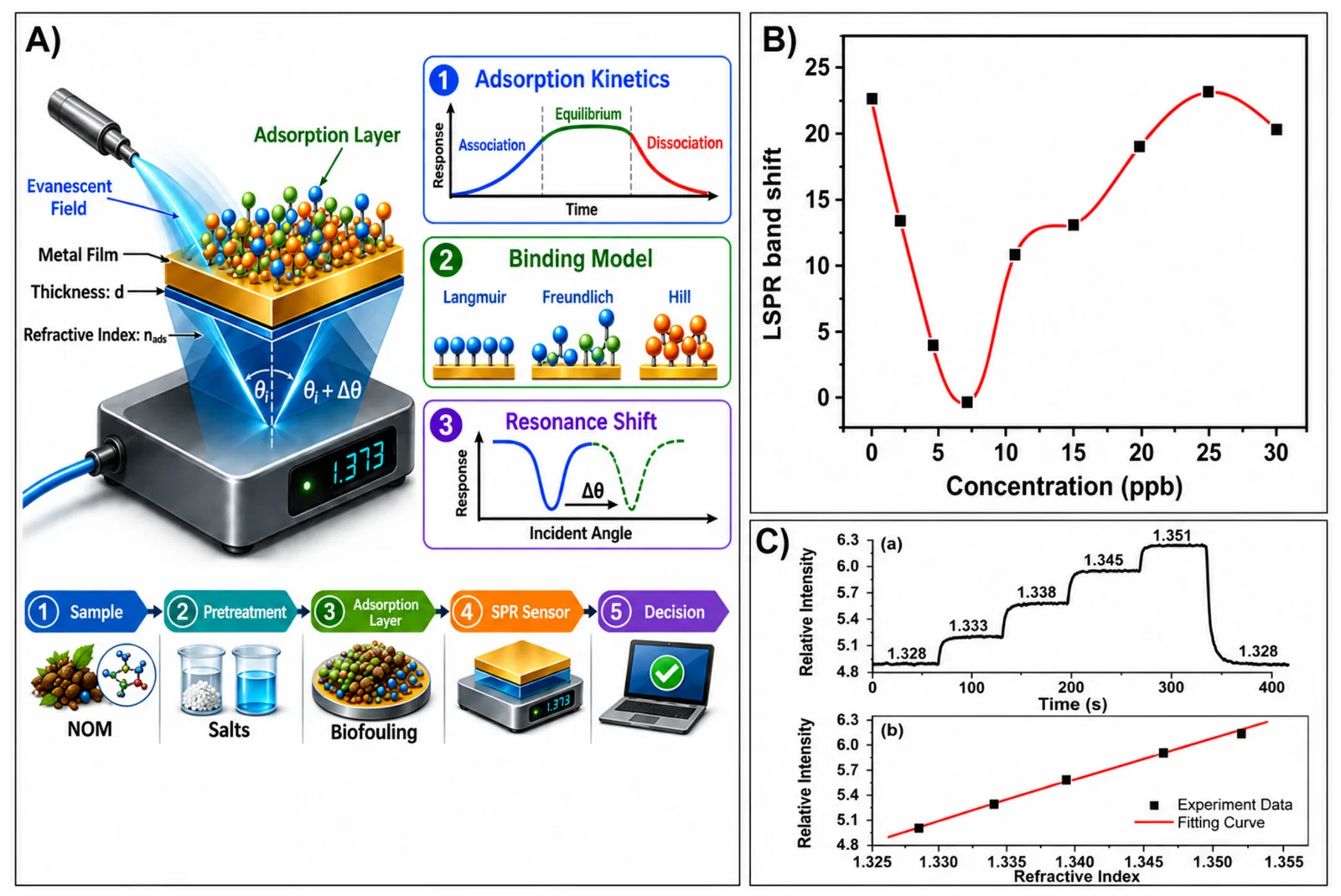
Plasmonic adsorption-based sensing principles and optical responses. (**A**) SPR adsorption mechanism showing p-polarized excitation and adsorption-induced resonance shifts. (**B**) LSPR band shifts for concentration calibration. (**C**) Smartphone-based SPR imaging sensor showing real-time and steady-state refractive-index responses. Panels (**B**,**C**) adapted from [18,19] CC-BY-4.0.

Future SPR platforms should move beyond simple resonance shift detection toward adsorption-engineered interfaces that combine high selectivity, regeneration, matrix tolerance, and long-term baseline stability. For environmental monitoring, the most important advances will come from robust surface chemistry, integrated sample pretreatment, and multiparameter readout, rather than from optical sensitivity alone. Figure 2A shows how adsorption within the evanescent field changes the effective refractive index near a metal film and shifts the SPR angle. The association, equilibrium, and dissociation stages can reveal binding behavior, although bulk refractive-index changes, transport, and nonspecific adsorption may also affect the trace. Reference channels and matrix-matched blanks are therefore needed to attribute a shift to the target. In Figure 2B, adsorption near a metal nanostructure changes its local dielectric environment and shifts the LSPR band. The plotted band shift varies nonmonotonically with concentration; interpret it as a measured calibration response rather than evidence of a universal linear relation. Aggregation, receptor saturation, and matrix effects may contribute and require experimental controls. Figure 2C illustrates a portable SPR imaging readout: stepwise intensity changes track solutions with different refractive indices, and the steady-state values provide a calibration. Stable illumination, image referencing, temperature control, and a selective adsorption layer are needed before assigning such a response to a specific environmental analyte.

### 2.2. Localized Surface Plasmon Resonance (LSPR): Nanoparticle Geometry, Local Environment, and Adsorption Sensitivity

LSPR arises from resonant electron oscillations in metallic nanostructures such as nanoparticles, nanorods, nanoantennas, and ordered arrays. Because the LSPR field is confined to the immediate nanoscale environment, adsorption within the decay length produces measurable changes in resonance wavelength, intensity, and linewidth [3,20,21,22,23]. This makes LSPR highly attractive for compact and miniaturized sensing platforms.

The practical advantage of LSPR is also its main limitation. Its very short sensing volume makes it extremely responsive to molecular adsorption and highly vulnerable to fouling, nonspecific binding, and local surface heterogeneity. Therefore, nanoparticle geometry, interparticle spacing, material composition, and coating thickness must be optimized together with surface chemistry. High-performance LSPR sensors should not be designed as isolated plasmonic structures; they should be treated as integrated nanostructure–interface systems in which target adsorption dominates the local dielectric response.

### 2.3. Surface-Enhanced Raman Scattering (SERS): Adsorption, Hot Spots, and Molecular-Level Fingerprints

SERS provides molecular fingerprinting and can identify adsorbed species through vibrational signatures. However, SERS depends more on adsorption than SPR and LSPR because the strongest signals occur only when molecules adsorb on plasmonic surfaces or within nanogap hot spots [24,25,26,27,28]. Electromagnetic enhancement arises from strongly localized fields, whereas chemical enhancement originates from adsorption-mediated charge transfer and changes in molecular polarizability.

The key conclusion is that molecular placement, binding orientation, surface charge, and hot-spot accessibility control SERS signal strength and spectral reproducibility. Competitive adsorption can block hot spots or introduce false spectral features, while different binding configurations can change peak ratios and vibrational selection rules. Therefore, SERS should be regarded not only as a sensitive detection method but also as a probe of adsorption configurations and interfacial chemistry.

Future SERS platforms should prioritize controlled hot-spot architecture, selective molecular capture, antifouling protection, quantitative calibration, and reproducible substrate fabrication. These requirements are essential for moving SERS from qualitative fingerprinting to reliable environmental analysis. Supplementary Figure S2 illustrates LSPR excitation in a noble-metal nanoparticle, in which incident light generates an enhanced near field. Adsorbed analytes experience amplified SERS signals and LSPR wavelength shifts, with the inset showing the plasmon-molecule energy coupling.

**Figure 3:**
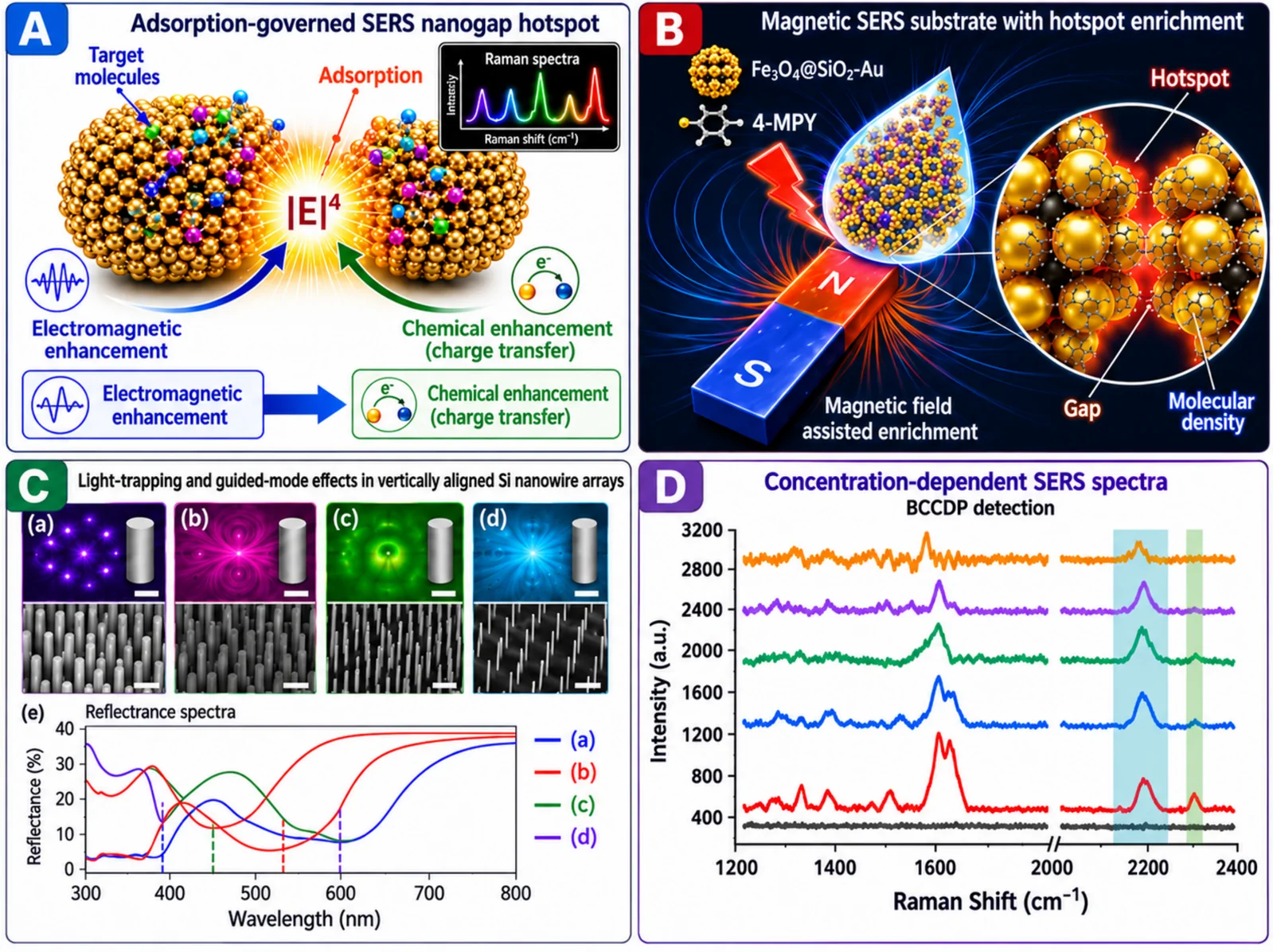
Adsorption-mediated SERS enhancement and light trapping. (**A**) SERS in nanogap hotspots, where molecular adsorption, orientation, charge transfer, and hotspot localization affect Raman sensitivity, selectivity, and reproducibility. (**B**) Magnetic Fe_3_O_4_@SiO_2_−Au SERS substrate showing magnetic enrichment, dense adsorption, and interparticle hotspot formation. Adapted from [29]. (**C**) Vertically aligned Si nanowire arrays showing diameter-dependent light trapping, reflectance, guided-mode absorption, electric-field distribution, and Fabry–Pérot oscillations. Adapted from [30,31] under CC BY 4.0. (**D**) Concentration-dependent SERS spectra for BCCDP detection, moved from the former (**C**); adapted from [32] under CC BY 4.0.

Figure 3 illustrates how adsorption, nanogap hotspots, magnetic enrichment, and guided optical modes jointly improve SERS performance by increasing local field intensity, molecular confinement, and light–matter interaction. In Figure 3A, a nanogap concentrates the excitation and Raman-scattered fields, but enhancement benefits an analyte only when adsorption places it inside the active region. Molecular orientation and competing adsorption can change both signal intensity and relative band strengths. Charge-transfer-mediated chemical enhancement may add to the electromagnetic contribution when the metal–molecule electronic structure permits it; it should not be assumed for every adsorbate. In Figure 3B, magnetic Fe_3_O_4_@SiO_2_–Au particles collect analytes and bring Au-rich surfaces together, increasing the likelihood of interparticle hot spots. Evaluate capture efficiency and electromagnetic enhancement separately. Figure 3C shows diameter-dependent reflectance and guided-mode behavior in Si nanowire arrays. These light-trapping effects can increase optical interaction, but the panel alone does not demonstrate SERS or adsorption; an appropriate plasmonic and capture interface would be needed for that application. The spectra in Figure 3D provide the distinct SERS readout. Concentration-dependent Raman peaks can support identification and calibration only after band assignment, blank subtraction, replicate measurements, and checks for matrix interference and hot-spot variability. The displayed traces alone do not establish a limit of detection.

Supplementary Table S2 summarizes the major adsorption mechanisms relevant to SPR, LSPR, and SERS, including physisorption, chemisorption, electrostatic and ion-exchange adsorption, charge-transfer interactions, competitive adsorption and fouling, and speciation-dependent binding, together with their effects on plasmonic signals and environmental sensing performance. Future LSPR platforms should prioritize reproducible arrays, narrow-linewidth resonators, antifouling layers, and multiplexed spectral analysis, since real-sample performance depends more on matrix tolerance, regeneration, and stable peak tracking than on buffer-based sensitivity.

In summary, SPR, LSPR, and SERS all rely on adsorption-induced changes at the plasmonic interface. SPR and LSPR transduce adsorption into resonance shifts, while SERS provides molecular fingerprinting through adsorption-mediated enhancement. The common theme is that adsorption efficiency, selectivity, and stability govern real-world performance more than optical sensitivity alone.

### 2.4. Theoretical Models and Simulation Tools: From Dielectric Functions to Adsorption-Aware Field Modeling

Mechanistic interpretation of plasmonic adsorption requires models that connect properties to measurable optical signals. Accurate dielectric functions provide the baseline optical response of metals and hybrid materials [33,34]. Mie-type models are useful for spherical or near-spherical particles and clarify how changes in the surrounding refractive index shift nanoparticle resonances [35]. The discrete dipole approximation (DDA) extends the modeling to irregular or complex nanostructures [36], while finite-difference time-domain (FDTD) and related full-wave methods are essential for nanogaps, multilayers, porous coatings, and realistic device geometries [37,38].

Optical simulation alone is insufficient for environmental sensing. Adsorption-aware modeling must include surface coverage, adsorption layer thickness, effective refractive index, molecular orientation, diffusion–convection transport, competitive binding, and fouling. Even simplified coupling of electromagnetic simulation with Langmuir/Freundlich isotherms, transport models, or multicomponent adsorption scenarios can greatly improve interpretation of resonance shifts, line-width changes, near-field maps, and SERS enhancement [39].

Supplementary Figures S3 and S4 show that plasmonic architectures, including colloidal nanoparticles, nanorod arrays, metasurfaces, and fiber probes, rely on analyte adsorption to modify plasmon–matter interactions and generate resonance shifts, near-field changes, and SERS enhancement. Future modeling should adopt multiscale, data-assisted frameworks that link adsorption parameters to optical outputs such as Δλ, Δθ, ΔI, linewidth, field intensity, and Raman enhancement, while using machine learning to distinguish target signals from drift, noise, nonspecific adsorption, and matrix interference.

## 3. Materials and Nanostructures for Plasmonic Adsorption

Plasmonic adsorption performance depends on the combined effects of material composition, nanostructure morphology, surface chemistry, and environmental stability. Noble metals such as Au and Ag remain the most effective platforms for strong LSPR, but practical deployment increasingly requires hybrid, selective, reusable, and sustainable material systems. The central design goal is no longer only to maximize electromagnetic enhancement; it is to create plasmonic interfaces that selectively adsorb target molecules, resist matrix interference, and generate reproducible optical signals under real environmental conditions [40,41].

The choice of material directly controls the resonant wavelength, field confinement, chemical compatibility, oxidation resistance, and functionalization strategy. Nanostructure design further determines hot-spot density, surface area, molecular accessibility, and signal stability. Therefore, evaluate plasmonic adsorption materials using both optical and interfacial criteria, including LSPR strength, adsorption selectivity, antifouling capability, regeneration, fabrication scalability, and environmental compatibility. Table 1 summarizes plasmonic (SPR, LSPR, SERS, colorimetric) platforms for environmental monitoring, detailing sample matrices, targets, recognition interfaces (e.g., aptamers, MIPs, MOFs), and performance. Supplementary Table S4 expands on readout modes, detection limits, and application benchmarks.

### 3.1. Noble Metals and Alloys

Shape control provides an additional tuning parameter: nanorods, nanocubes, nanoplates, nanostars, and nanoporous architectures can be designed to place the resonance near the excitation or detection wavelength and to increase the hot spot density for adsorption-enhanced sensing [3]. Au and Ag nanoparticles remain the benchmark materials for plasmonic adsorption because they support intense LSPRs in the visible and near-infrared regions and provide strong local-field enhancement [50,51]. Au offers chemical stability, biocompatibility, and well-established thiol-based surface chemistry, making it highly suitable for biosensing, heavy-metal detection, and receptor-functionalized interfaces. Ag provides stronger plasmonic enhancement and sharper resonances, which are advantageous for SERS, but its practical use is limited by oxidation, sulfidation, and reduced long-term stability in complex environments.

The key conclusion is that researchers should select noble metals based on the dominant requirement. Ag-rich nanostructures are preferred for maximum electromagnetic enhancement, whereas Au-rich platforms are more reliable when chemical stability, biofunctionalization, and reproducibility are critical.

Alloy and core–shell structures provide a practical route to balance sensitivity and stability. Au–Ag alloys allow continuous LSPR tuning while improving stability relative to pure Ag, whereas Au@Ag or Ag@Au core–shell designs combine strong field enhancement with surface protection and tailored chemistry [52,53,54]. Future noble metal platforms should emphasize oxidation-resistant Ag hybrids, low-cost alloy compositions, reproducible hot-spot architectures, and surface coatings that maintain adsorption selectivity during repeated use. In this direction, noble metals will remain important not as standalone materials but as active plasmonic cores within more robust multifunctional architectures.

### 3.2. Hybrid Nanostructures and Two-Dimensional Materials

Hybrid nanostructures expand plasmonic adsorption beyond the limitations of single-component metals. Metal–semiconductor hybrids, such as Au/TiO_2_, Ag/TiO_2_, Au/ZnO, and Ag/ZnO, combine strong plasmonic light concentration with semiconductor adsorption sites, charge separation, and photocatalytic activity [55]. In these systems, the metal component acts as an optical antenna, while the semiconductor contributes to chemical affinity, charge-mediated adsorption, and reaction pathways. This architecture is particularly promising for pollutant sensing, photocatalytic degradation, gas detection, and light-assisted environmental remediation.

Two-dimensional materials provide another important direction for adsorption-enhanced plasmonics. Graphene, graphene oxide, MoS_2_, WS_2_, MXenes, and related layered materials offer high surface area, tunable surface chemistry, and strong interactions with organic molecules, gases, and biomolecules [56,57,58,59,60]. When combined with Au or Ag nanoparticles, these materials improve analyte enrichment near plasmonic hot spots and suppress nanoparticle aggregation. Graphene-based hybrids are especially useful for aromatic pollutants because π–π interactions promote adsorption, while metal nanoparticles enhance LSPR or SERS. MoS_2_ and other transition-metal dichalcogenides can introduce defect-mediated binding, charge transfer, and analyte-specific adsorption, thereby enabling improved gas and chemical detection.

The main future opportunity lies in the rational design of heterostructures. Hybrid materials should be engineered so that the plasmonic component provides field enhancement, the semiconductor or two-dimensional (2D) layer provides selective adsorption and charge transfer, and the overall architecture remains stable, reusable, and scalable. Future research should prioritize interface-controlled synthesis, defect engineering, hotspot placement, and standardized testing on real matrices. Hybrid nanostructures are expected to become central to plasmonic adsorption technologies because they can combine sensitivity, selectivity, photocatalytic functionality, and environmental robustness on a single platform.

### 3.3. Functionalization and Molecularly Imprinted Polymers

Surface functionalization is essential for transforming plasmonic nanostructures from highly sensitive optical materials into selective adsorption-based sensing interfaces. Bare Au and Ag surfaces can interact with a broad range of adsorbates, which may lead to nonspecific responses in complex samples. Therefore, researchers commonly introduce functional coatings and molecular recognition elements, including self-assembled monolayers, silanes, polymers, antibodies, DNA/DNAzymes, aptamers, enzymes, porous frameworks, and molecularly imprinted polymers (MIPs), to control target adsorption and position analytes within the plasmonic near field [61,62].

Antibody-functionalized plasmonic interfaces provide highly specific antigen recognition and are widely used in SPR and LSPR biosensors, where antigen–antibody binding increases the effective refractive index near the metal surface and produces measurable resonance-angle or wavelength shifts. Antibodies can also be incorporated into SERS-based assays by capturing substrates or Raman nanotags, enabling selective enrichment of target biomolecules near electromagnetic hot spots. However, antibody-based interfaces may suffer from limited storage stability, orientation-dependent activity, and nonspecific protein adsorption, particularly in complex sample matrices.

Nucleic-acid recognition provides another important strategy for adsorption-controlled plasmonic sensing. DNA probes can selectively capture complementary sequences through hybridization, whereas DNAzymes can provide ion-specific recognition. When immobilized on Au nanoparticles or plasmonic films, target-induced hybridization, cleavage, conformational change, or nanoparticle aggregation can generate SPR/LSPR wavelength shifts or colorimetric responses. DNA-based nanostructures can additionally organize plasmonic nanoparticles with nanoscale precision, thereby creating controlled electromagnetic hot spots for SERS enhancement. Such approaches demonstrate the close relationship between molecular adsorption, nanoscale organization, and plasmonic signal generation.

Aptamers offer an alternative class of recognition molecules with high affinity for ions, proteins, toxins, pathogens, and other molecular targets. Aptamer-functionalized SPR and LSPR platforms translate target-induced binding or conformational changes into resonance shifts, whereas aptamer-assisted SERS platforms can selectively concentrate analytes within Raman-active hot spots. For example, antibody–aptamer plasmonic configurations have demonstrated highly sensitive biomarker detection, illustrating the potential of combining complementary recognition mechanisms within a single plasmonic interface. Compared with antibodies, aptamers can offer advantages such as chemical synthesis, relatively high stability, facile modification, and regeneration, although their binding performance can remain sensitive to ionic strength, pH, and sample composition.

Molecularly imprinted polymers (MIPs) provide a complementary synthetic-recognition approach [7,63,64]. MIPs contain target-shaped binding cavities produced using template molecules and can therefore achieve selective rebinding through molecular size, shape, and functional-group complementarity [65,66]. When integrated with SPR, LSPR, or SERS substrates, adsorption into these imprinted sites changes the local refractive index, plasmonic coupling, or Raman intensity. Compared with many biological receptors, MIPs can provide improved chemical stability, reusability, and resistance to harsh environmental conditions. Collectively, antibodies, DNA/DNAzymes, aptamers, and MIPs demonstrate that the performance of adsorption-based plasmonic sensors depends not only on the constituent plasmonic material but also on the design of the recognition layer that selectively captures and positions target analytes within the optically active sensing region [67].

### 3.4. Emerging Biocompatible and Sustainable Materials

The transition from laboratory demonstrations to large-scale environmental deployment requires plasmonic materials that are not only sensitive but also low-cost, stable, scalable, and environmentally responsible. Au and Ag offer excellent optical performance, but their cost, scarcity, and long-term environmental concerns motivate the development of alternative plasmonic platforms [68]. Therefore, sustainable plasmonics should be considered a core design requirement, especially for disposable sensors, large-area monitoring networks, and remediation devices.

Al and Cu are promising low-cost alternatives for selected wavelength ranges. Al supports plasmonic resonances across the ultraviolet-to-visible regions and is compatible with scalable semiconductor processing, while its native oxide can provide partial protection and surface functionality. Cu offers lower cost and a visible plasmonic response but requires stabilization against oxidation via coatings, alloying, or encapsulation [69,70]. Doped oxides, such as indium tin oxide (ITO) and Aluminum-doped Zinc Oxide (AZO), as well as transition metal nitrides, such as TiN and ZrN, further expand the material toolbox by providing thermal stability, complementary metal-oxide-semiconductor (CMOS) compatibility, and operation in harsh environments [71,72].

Supplementary Table S3 further summarizes representative emerging plasmonic materials and their associated fabrication strategies, including refractory metal nitrides, doped transparent oxides, topological insulators, two-dimensional materials, hybrid metal–dielectric metasurfaces, and flexible plasmonic substrates.

Biodegradable and bio-derived substrates provide another sustainable pathway. Cellulose, bacterial nanocellulose, gelatin, paper, and biopolymer membranes can host plasmonic nanoparticles or thin metal films to form low-cost, flexible, and disposable sensor platforms [73,74,75]. Such substrates are particularly valuable for field testing, point-of-use diagnostics, and environmental monitoring in sensitive ecosystems. Magnesium and other biodegradable plasmonic materials may also enable transient or single-use sensors, although oxidation control remains a key challenge [76].

**Figure 4:**
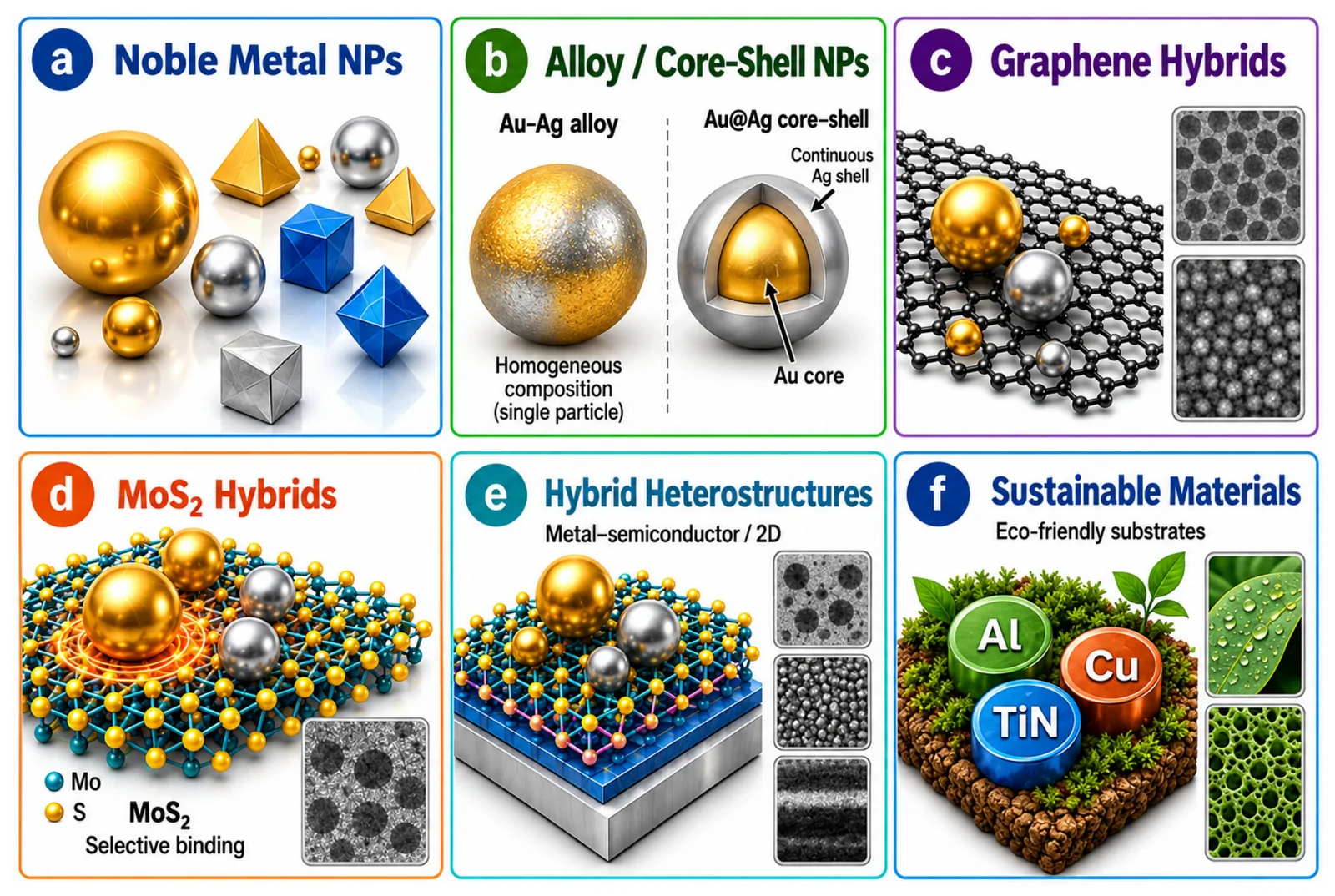
Plasmonic material platforms for adsorption-based sensing. Representative noble-metal, alloy/core–shell, graphene, MoS_2_, metal-semiconductor/2D hybrid, and sustainable plasmonic materials for improving adsorption, selectivity, stability, and optical response. Core–shell panel redrawn to show a single core particle with a continuous shell layer. Adapted from [77] CC-BY-4.0.

Future plasmonic adsorption platforms should integrate green materials, low-waste fabrication, recyclable components, and end-of-life considerations from the design stage. Sustainable materials will be more impactful when combined with selective surface chemistry, scalable nanomanufacturing, and stable optical performance. Figure 4 compares materials by the functions of their exposed interfaces, not simply by resonance brightness. Au enables stable thiol-based anchoring, whereas Ag often offers stronger local fields but can oxidize or sulfidize in environmental matrices. In a core–shell particle, a continuous shell encloses one core: its thickness determines which material contacts the sample, how effectively analytes approach the enhanced field, and how well it protects the core. This differs from nanoparticles deposited on a support, where gaps and exposed support sites govern adsorption. Graphene and MoS_2_ hybrids can enrich adsorbates or provide charge-transfer pathways, but adding a layer can also separate targets from the metal near field. The optical benefit of a hybrid should therefore be assessed together with competitive adsorption, chemical stability, and reproducibility; alternative metals and substrates require the same interface-level tests.

Supplementary Figure S5 summarizes Langmuir/Freundlich adsorption isotherms and pseudo-first- and second-order kinetic models, illustrating how adsorption capacity, surface heterogeneity, and adsorption rate influence plasmonic sensing signals, such as spectral shifts and intensity changes.

For translational comparison, benchmark plasmonic sensing platforms using a common analytical framework. Beyond the detection limit and linear range, key performance descriptors should include matrix type, pretreatment requirements, response time, regeneration capability, storage stability, batch-to-batch reproducibility, reference-method validation, and readiness for portable deployment. A low nominal detection limit obtained in buffer should not be interpreted as superior to a slightly higher detection limit reproducibly obtained in untreated or minimally processed environmental samples.

## 4. Plasmonic Adsorption in Environmental Monitoring

Plasmonic adsorption-based sensing provides a promising route for rapid, sensitive, and field-deployable environmental monitoring. Its value lies in converting contaminant adsorption at engineered plasmonic interfaces into optical signals, such as color changes, resonance shifts, intensity variations, or Raman fingerprints [78,79]. However, the practical success of these sensors depends not only on plasmonic enhancement but also on selective adsorption, matrix tolerance, signal stability, and integration with portable readout systems. Therefore, environmental plasmonic sensors should be developed as complete analytical platforms that combine sample handling, adsorption control, optical transduction, and data interpretation.

### 4.1. Water Quality Monitoring

Water monitoring is one of the most important applications of plasmonic adsorption because many contaminants, including heavy metals, pesticides, pharmaceuticals, pathogens, and toxins, must be detected rapidly at trace levels [80]. Plasmonic sensors can address this need by using functionalized Au or Ag nanostructures to selectively adsorb target species and translate binding into LSPR shifts, SPR angle changes, colorimetric responses, or SERS fingerprints.

Heavy metal detection is a representative example. Ligand-, DNAzyme-, thiol-, or chelator-functionalized plasmonic nanoparticles can capture ions such as Hg^2+^, Pb^2+^, Cd^2+^, and As^3+^, producing nanoparticle aggregation, spectral shifts, or mass-sensitive optical changes [81,82,83]. These systems can reach very low detection limits because even small adsorption events strongly perturb the local plasmonic environment. For organic pollutants such as pesticides and pharmaceutical residues, SERS is particularly useful because it provides molecular fingerprints rather than only refractive index changes [84,85]. Pathogens and toxins can also be detected via antibody-, aptamer-, or receptor-modified plasmonic surfaces, enabling faster readout than conventional culture-based methods [86,87,88].

The key conclusion is that water sensing performance is limited mainly by matrix interference and surface fouling rather than by optical sensitivity alone. Natural organic matter, salts, suspended particles, and biofilms can compete with target analytes, block active sites, and distort baselines. Therefore, future water quality sensors should integrate antifouling coatings, selective receptors, microfluidic pretreatment, nanofiltration, and internal referencing. These improvements will be essential to transform laboratory plasmonic assays into reliable real-time water surveillance tools.

### 4.2. Air and Gas Sensing

Plasmonic gas sensors exploit adsorption-induced changes in refractive index, electron density, molecular absorption, or plasmon–molecule coupling to detect gaseous pollutants under ambient conditions [89]. Compared to conventional electrochemical or metal-oxide gas sensors, plasmonic systems can offer low-power optical readout, fast response, and compatibility with remote or distributed monitoring.

Several architectures are promising. LSPR sensors based on functionalized nanoparticles, nanodisks, nanoholes, or MOF-coated plasmonic structures can detect gases such as NO_2_, NH_3_, benzene, and toluene by concentrating gas molecules near the plasmonic surface [6,90]. Plasmonic perfect absorbers and metasurfaces provide another route by aligning narrow optical resonances with molecular absorption bands, thereby enabling compact infrared gas detection without bulky spectrometers [44,91,92]. SERS-based gas sensors are useful for VOCs and hazardous vapors when capture coatings enrich molecules at plasmonic hot spots [93]. Hybrid chemoplasmonic systems, such as metal–oxide/plasmonic nanostructures, further improve selectivity by combining chemical reaction sites with optical transduction [94,95].

The main challenge for plasmonic gas sensing is selectivity under variable humidity, temperature, and gas-mixture conditions. Adsorption coatings must concentrate the target gas while rejecting interferences and allowing regeneration. Future systems should combine plasmonic sensor arrays, selective porous coatings, microheaters, humidity correction, and machine-learning-based pattern recognition. Such integrated platforms could support urban air quality networks, industrial leak detection, greenhouse gas monitoring, and wearable exposure badges [96].

### 4.3. Detection of Soil and Food Contaminants

Soil and food monitoring require rapid methods that can operate in complex matrices with minimal sample preparation. Plasmonic adsorption sensors are attractive because they can detect pesticides, heavy metals, mycotoxins, antibiotics, hormones, pathogens, and spoilage markers using compact optical readouts [97]. However, unlike water or gas samples, soil and food often require extraction, swabbing, dilution, or surface sampling before analytes can reach the plasmonic interface.

For soil analysis, plasmonic assays have been used to detect heavy metals and persistent organic pollutants in leachates or extracts [98,99]. Colorimetric Au nanoparticle systems can quantify metal ions through aggregation-induced spectral changes, while SERS substrates can identify organic contaminants transferred from the soil to plasmonic surfaces. For food safety, paper-based SERS strips, plasmonic immunosensors, and SPR biosensors have enabled the detection of pesticide residues, aflatoxins, antibiotics, allergens, and microbial toxins [100,101,102]. Plasmonic platforms are also being explored for nutrient detection, such as nitrate and phosphate detection, which could support field-based agricultural management [103,104,105].

In conclusion, soil and food applications require more attention to sampling and interface selectivity than to optical enhancement alone. Reliable field operation will depend on simple extraction protocols, plasmonic swabs, disposable test strips, molecularly imprinted polymers, antifouling coatings, and handheld readers. Future research should prioritize matrix validation, recovery efficiency, reproducibility, and user-friendly workflows so that plasmonic detection can serve farmers, inspectors, and consumers as a practical screening tool.

### 4.4. Portable and Point-of-Care Systems

The strongest translational advantage of plasmonic adsorption sensors is their compatibility with portable and point-of-care systems. Plasmonic readouts can be miniaturized using LEDs, laser diodes, photodiodes, compact spectrometers, smartphone cameras, and disposable sensor chips [106,107,108]. This makes it possible to perform environmental tests outside centralized laboratories.

Smartphone-based plasmonic sensors are particularly promising because they combine imaging, illumination, computation, connectivity, and user interface on a single platform [109,110].

A plasmonic strip, SPR chip, or colorimetric nanoparticle assay can be coupled to a phone camera to quantify color intensity, spectral shifts, or resonance changes. Dedicated handheld devices can further improve reliability by integrating optical alignment, microfluidics, reference channels, and preloaded calibration models [111]. Portable systems are already being explored for water pollutant monitoring, pesticide residue detection, gas monitoring, and food safety testing.

Future portable systems should move beyond simple detection toward intelligent sensing. Cloud connectivity and machine learning can improve multianalyte recognition, drift correction, noise removal, and complex spectra [112,113]. Rugged packaging, reference channels, low-power operation, disposable cartridges, and automated calibration will be essential for field use [114].

**Figure 5:**
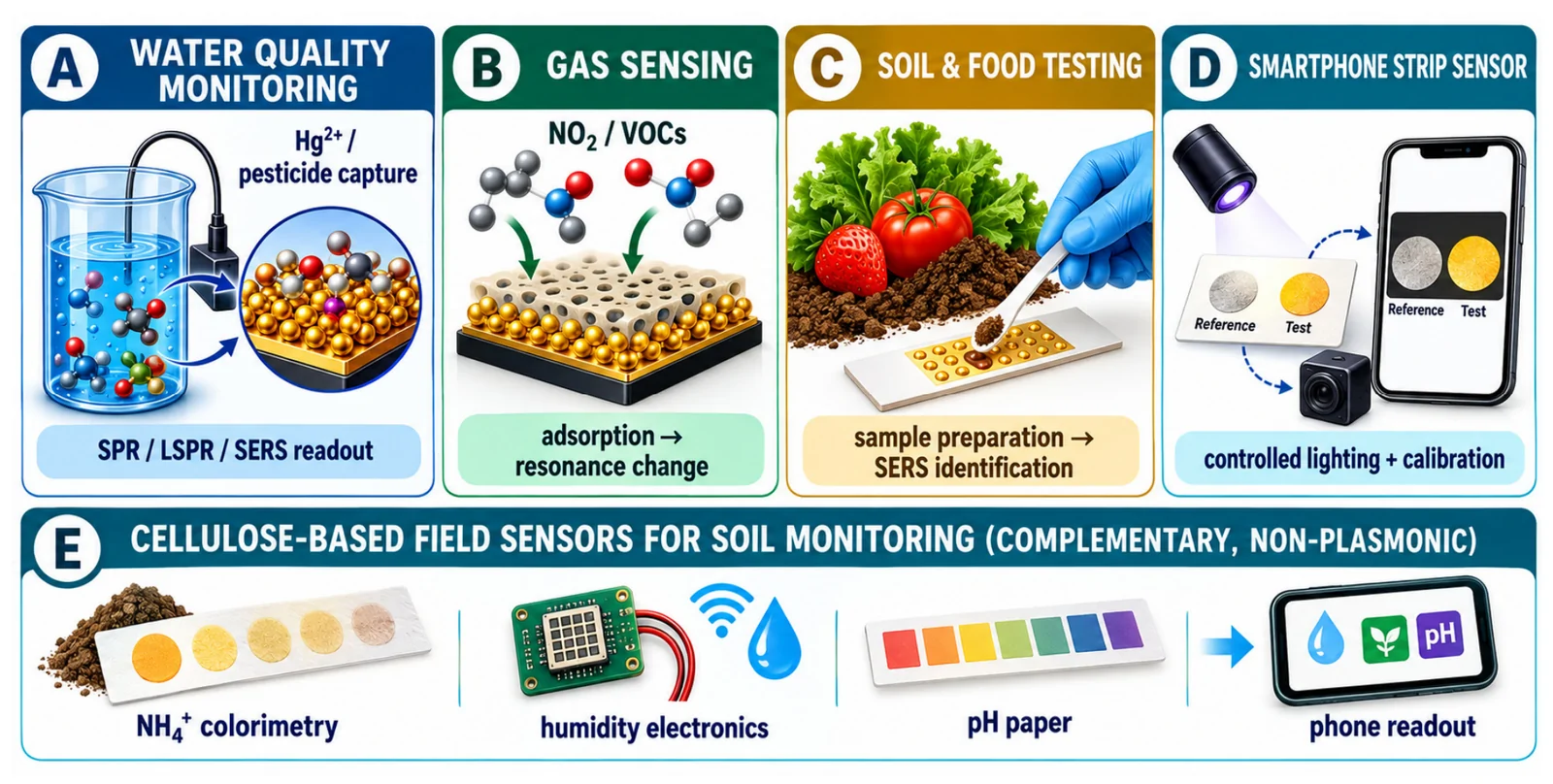
Plasmonic and cellulose-based sensors for environmental monitoring. (**A**–**D**) Plasmonic platforms for water-quality analysis, gas detection, soil/food testing, and smartphone-integrated disposable sensing, where contaminant adsorption produces optical resonance, SERS, or intensity changes. (**E**) Cellulose-based soil sensors for NH_4_^+^, humidity, and pH monitoring using impedance, wireless, and smartphone-assisted colorimetric readouts. Images adapted from [115,116] under CC-BY 4.0.

Table S4 highlights emerging plasmonic materials beyond Au and Ag for stable, scalable, flexible, and field-compatible sensors. Figure 5 presents plasmonic and cellulose-based sensing platforms for environmental monitoring. The top panels of Figure 5 represent different sample-to-signal chains. In water, dissolved ions and natural organic matter compete for recognition sites and change the bulk refractive index; in air, a porous capture layer may preconcentrate a gas while humidity alters its uptake. Soil and food measurements first require extraction or swabbing, so incomplete analyte recovery can limit accuracy even when the plasmonic transducer is highly sensitive. A phone or disposable strip provides convenient readout only after optical referencing, matrix-matched calibration, and interference controls. Panel E illustrates related cellulose-supported humidity and pH measurements; those electrical and colorimetric examples are not, by themselves, plasmonic adsorption readouts. Their relevance is the low-cost sampling, packaging, and digital-reporting workflow a plasmonic device could adopt. Claims of field suitability should report recovery, drift, between-device variation, and comparison with a reference method. Supplementary Figure S6 describes the field workflows from sampling and pretreatment to plasmonic detection. Overall, future environmental monitoring should prioritize selective interfaces, antifouling design, portable readout, robust calibration, and intelligent data analysis.

## 5. Broader Applications of Plasmonic Adsorption

Plasmonic adsorption extends beyond environmental sensing. The same interfacial mechanisms that support pollutant detection—localized field enhancement, hot carrier generation, photothermal conversion, and adsorption-mediated charge transfer—also advance biosensing, medical diagnostics, photocatalysis, energy conversion, optical data storage, computing, and environmental remediation. In these applications, plasmonic nanostructures primarily concentrate light and energy at adsorbed molecules or nearby functional materials. Thus, plasmonic adsorption should be viewed as a broad interface-engineering strategy for detection and light-driven transformation.

### 5.1. Biosensing and Biomedical Diagnostics

The following section briefly summarizes non-environmental application domains to illustrate the transferability of adsorption-governed plasmonic principles. Because this review focuses on environmental sensing, these examples are discussed comparatively rather than exhaustively.

Plasmonic nanostructures have become powerful biosensing platforms because they convert biomolecular adsorption into highly sensitive optical signals. In SPR and LSPR biosensors, proteins, nucleic acids, pathogens, cancer biomarkers, or cells are captured on functionalized Au or Ag surfaces, producing resonance shifts that can be monitored in real time [117]. This label-free capability is especially valuable for studying antigen–antibody binding, DNA hybridization, receptor–ligand interactions, and disease-related biomarkers.

The key advantage of plasmonic biosensing is its ability to detect small adsorption events in extremely small volumes. Nanodisks, nanoholes, nanoparticle arrays, and nanogap structures confine electromagnetic fields near the sensing interface, thus enabling enhanced detection of low-abundance biomolecules [118]. SERS nanotags and plasmon-enhanced fluorescence further extend sensitivity by amplifying Raman or fluorescence signals from target-bound probes [119,120,121]. These strategies are important for early diagnosis, where biomarkers may appear at the femtomolar, attomolar, or single-molecule levels.

Plasmonic nanoparticles also serve as diagnostic agents in imaging and in vivo analysis. Functionalized Au nanoparticles can selectively adsorb or bind to tumors, plaques, pathogens, or disease markers, allowing photoacoustic imaging, photothermal imaging, or SERS-based tissue identification [122]. Integration with microfluidics and portable optical readers further supports point-of-care diagnostics using small sample volumes, such as blood, saliva, or urine [123]. Adsorption of the target (human immunodeficiency virus (HIV) p24 antigen) onto the sensor, followed by binding of a Au nanoparticle label, produced a strong LSPR shift detectable by a miniature spectrometer. This demonstrates how plasmonic adsorption can enable high-end laboratory diagnostics to be delivered directly to patients.

The main future challenge is no longer sensitivity alone, but clinical reliability. Plasmonic biosensors must address nonspecific adsorption, protein corona formation, batch-to-batch variability, biological matrix effects, and long-term stability. Future systems should combine antifouling interfaces, standardized biofunctionalization, multiplexed detection, microfluidic sample preparation, and machine learning-assisted signal interpretation. These advances will determine whether plasmonic biosensors can move from laboratory demonstrations to routine clinical and point-of-care diagnostics.

### 5.2. Photocatalysis and Energy Conversion

Plasmonic adsorption plays a central role in photocatalysis and energy conversion because reactant molecules must first adsorb at active interfaces before light-driven charge transfer or thermal activation can occur. Under illumination, plasmonic metals such as Au, Ag, and Al can generate intense near-fields, hot electrons, hot holes, and localized heat. These effects can activate adsorbed molecules and accelerate reactions such as water splitting, CO_2_ reduction, pollutant degradation, selective oxidation, and solar thermocatalysis [50,124].

In metal–semiconductor hybrids such as Au/TiO_2_, Ag/TiO_2_, Au/ZnO, or plasmonic catalyst support systems, the plasmonic component acts as an optical antenna, while the semiconductor or catalytic component provides adsorption and reaction sites. Hot carrier injection can extend semiconductor photocatalysis into the visible range, while local photothermal heating can accelerate reaction kinetics, promote product desorption, and reduce surface poisoning. This dual action is especially useful for solar-driven pollutant degradation, hydrogen generation, CO_2_ conversion, and green chemical synthesis.

Plasmonic structures also improve solar energy harvesting by increasing light trapping, near-field coupling, and absorption in photovoltaic or photoelectrochemical materials. In dye-sensitized, perovskite, silicon, and thin-film solar cells, plasmonic nanoparticles can enhance the photocurrent by scattering light or intensifying local fields near absorbers. In ‘antenna-reactor’ catalysts, the antenna concentrates optical energy, while the reactor site converts adsorbed molecules into desired products.

The main conclusion is that plasmonic photocatalysis is most effective when adsorption, charge transfer, and light management are designed together. Future progress should focus on reaction selectivity, catalyst durability, hot carrier utilization efficiency, scalable synthesis, and operation under real solar illumination rather than idealized laboratory conditions. If these challenges are addressed, plasmonic adsorption could contribute to solar fuels, low-energy chemical manufacturing, and sustainable pollutant conversion.

### 5.3. Environmental Remediation

Plasmonic adsorption can also remove, degrade, or neutralize contaminants, extending its role from detection to active remediation. In these systems, pollutants adsorb first onto plasmonic or hybrid catalyst surfaces, where near-field enhancement, hot carriers, or photothermal heating drive chemical degradation, sterilization, or separation [125]. This creates a direct connection between sensing and treatment: the same adsorption interface that detects pollutants can also help eliminate them.

In water treatment, plasmonic photocatalysts such as Au/TiO_2_, Ag/TiO_2_ and other metal–semiconductor hybrids can accelerate the degradation of dyes, pesticides, pharmaceuticals, phenols, and persistent organic pollutants under visible or solar light [126,127].

Supplementary Figure S7 highlights four applications of plasmonic adsorption: biosensing, photocatalysis/energy conversion, data storage/computing, and environmental remediation, demonstrating its broad impact across diagnostics, energy, information technology, and environmental protection.

The plasmonic component improves light absorption and charge generation, while the catalyst surface adsorbs pollutants and facilitates radical formation. Solar steam generation provides another route in which plasmonic absorbers convert sunlight into localized heat for desalination, disinfection, and water purification [128,129].

For air remediation, plasmonic photocatalytic filters can capture volatile organic compounds, formaldehyde, pathogens, and other airborne pollutants and degrade them under illumination [130]. In soil remediation, plasmonic photocatalysts may facilitate in situ degradation of persistent pollutants, although particle transport, recovery, and ecological safety remain major concerns [131]. Emerging work on microplastics further suggests that plasmonic nanostructures can detect polymer fragments through SERS and promote their photochemical degradation under light [45].

Supplementary Figure S8 summarizes plasmonic remediation strategies for water, air, soil, and microplastics, where adsorption, near-field enhancement, photothermal effects, and hot-carrier transfer enable degradation, sterilization, and purification. Future work should prioritize stable, recoverable, reusable, and nontoxic materials for sunlight-driven operation in complex environments, supported by immobilized catalysts, reusable membranes, flow-through reactors, scalable fabrication, data analysis, and life-cycle assessment to avoid secondary nanoparticle pollution.

In summary, plasmonic adsorption extends beyond environmental sensing to biosensing, photocatalysis, energy conversion, data storage, and environmental remediation. The common theme is that adsorption at the plasmonic interface governs interactions among light, molecules, and functional materials.

## 6. Challenges and Limitations

Despite rapid progress, plasmonic adsorption-based sensors still face major barriers to practical deployment. The most important limitations are not only optical sensitivity but also selectivity, matrix tolerance, long-term stability, fabrication reproducibility, scalability, and regulatory validation. Many systems achieve excellent detection limits under controlled laboratory conditions, but their performance often decreases in complex environmental or biological samples. Therefore, future development must shift from isolated performance demonstrations to complete sensing platforms that are stable, reproducible, standardized, and validated under realistic operating conditions.

### 6.1. Sensitivity, Selectivity, and Limit of Detection

The limit of detection (LOD) of plasmonic sensors is ultimately governed by the SNR, not by sensitivity alone. In ideal samples, the SPR, LSPR, and SERS platforms can detect very small adsorption events; however, real samples contain salts, proteins, natural organic matter, surfactants, particles, and other species that can nonspecifically adsorb, scatter light, or foul the plasmonic surface [40]. These matrix effects increase background noise, distort baselines, and reduce the reproducibility of ultralow LODs reported in purified systems.

Selectivity remains a central challenge, because bare plasmonic surfaces respond to many adsorbed species. A resonance change, a color change, or a Raman signal can arise from the target analyte, an interference, or nonspecific binding [132]. Therefore, selective receptors such as antibodies, aptamers, thiol ligands, molecularly imprinted polymers, and ion-recognition layers are essential. However, recognition layers can also reduce sensitivity by increasing the distance between the analyte and the plasmonic field or by blocking active hot spots [133]. The key design challenge is to balance selective adsorption with strong field overlap.

Instrumental noise also limits detectability. Photon shot noise, detector noise, thermal drift, laser intensity fluctuation, resonance linewidth, and structural non-uniformity define the minimum measurable shift or intensity change [134]. Referencing strategies, differential channels, modulation, lock-in detection, and advanced signal processing can improve measurement precision [135]. Coupled plasmonic–photonic modes, surface lattice resonances, and high Q hybrid structures may further improve detectivity when sensitivity and linewidth are optimized together.

**Figure 6:**
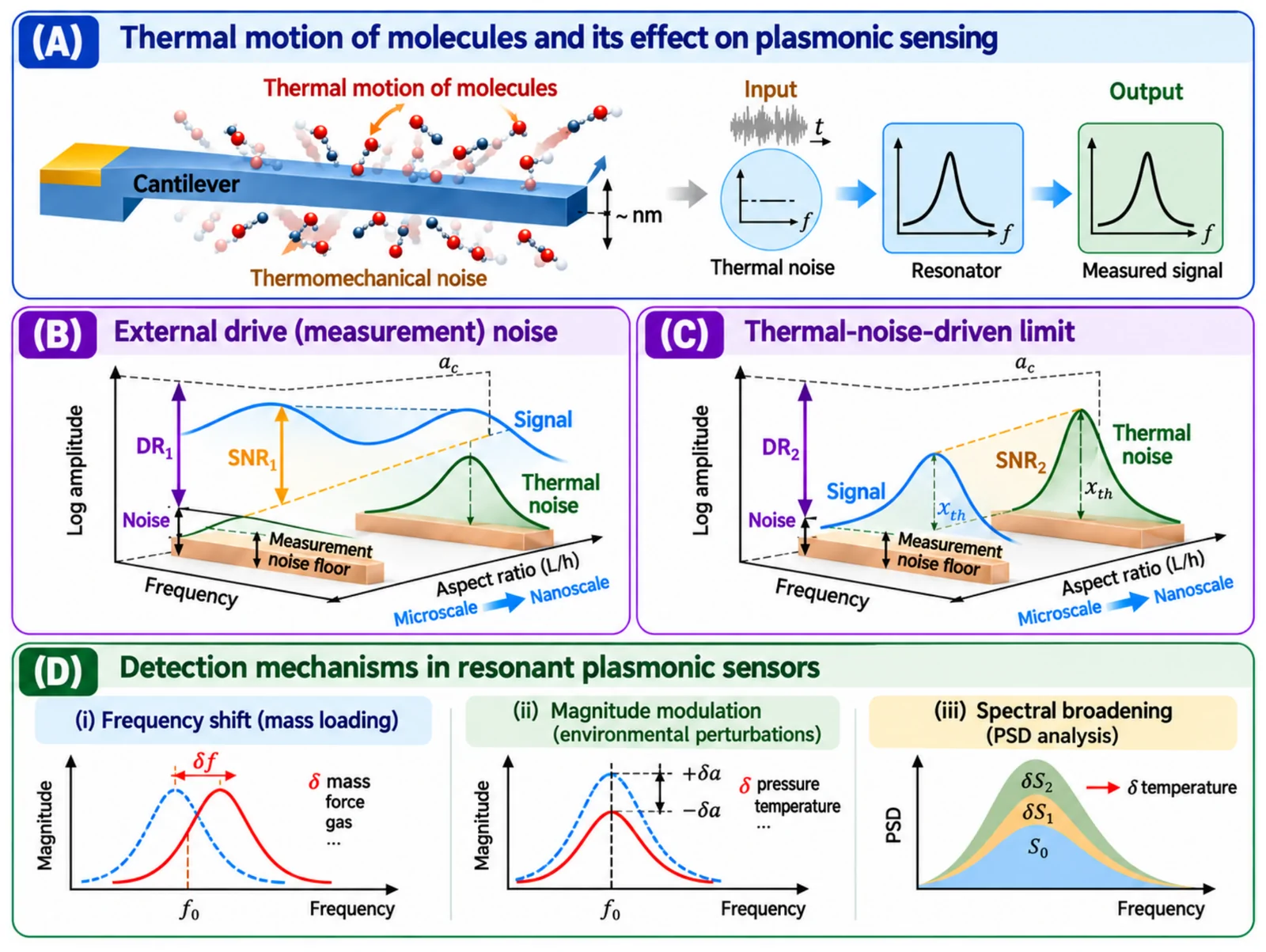
Noise and SNR principles in resonance sensors. (**A**) Schematic illustration of thermomechanical noise in a cantilever resonator, showing how thermal input fluctuations convert into a measurable resonator output. (**B**) Externally driven operation, highlighting the relationship among signal, noise, dynamic range, and signal-to-noise ratio (SNR). (**C**) Thermal-noise-driven operation, illustrating the thermally limited detection regime and its influence on dynamic range and SNR. (**D**) Detection mechanisms in resonant plasmonic sensors, including (**i**) frequency shift, (**ii**) peak/magnitude modulation, and (**iii**) power spectral density (PSD) variation. Panels (**A**–**D**) adapted from [136] CC-BY 4.0.

Future sensors should be designed around SNR engineering. This includes antifouling coatings such as polyethylene glycol, zwitterionic polymers, and hydrophilic brushes; selective receptors that suppress cross-reactivity; nanostructures that localize fields in reproducible hot spots; and multiplexed sensor arrays that provide pattern-based identification. Machine learning can also help distinguish target signals from drift, matrix effects, and nonspecific adsorption. Figure 6 connects practical environmental plasmonic sensing platforms with fundamental noise-limited detection concepts, emphasizing how adsorption response, SNR, dynamic range, and resonance stability govern real-world sensor performance.

Figure 6A–D illustrates general resonance-sensor concepts related to noise floor, signal-to-noise ratio (SNR), dynamic range, and detection mechanisms. The thermomechanical-noise and frequency-shift schematics are conceptual representations of resonance-limited detection, not direct measurements of the plasmonic devices discussed in this review. For optical SPR and LSPR sensors, the smallest resolvable resonance shift depends not only on nominal sensitivity but also on resonance linewidth, spectral-fitting accuracy, illumination stability, detector fluctuations, temperature variations, and baseline drift. These factors collectively define the effective noise floor and therefore the practical limit of detection. For SERS platforms, additional variability arises from hot-spot heterogeneity, spatial nonuniformity of analyte adsorption, substrate-to-substrate variation, and spectral background, all of which can influence signal reproducibility and quantitative performance. Accordingly, reliable environmental detection requires appropriate referencing and validation strategies, including reference channels, replicate sensing chips, spatial mapping when relevant, and clearly defined blank-based decision criteria. Such controls are essential to distinguish target-induced signals from instrumental drift, nonspecific adsorption, and matrix-related fluctuations before interpreting a laboratory response as a credible environmental detection event.

### 6.2. Stability and Biocompatibility

Chemical, physical, and biological stability are essential for plasmonic sensors intended for continuous monitoring, repeated use, or deployment in environmental and clinical settings. Many plasmonic structures rely on Au, Ag, Cu, or nanostructured films, which can undergo oxidation, sulfidation, aggregation, corrosion, dissolution, or surface contamination over time [137]. Ag provides strong plasmonic enhancement, but is prone to tarnishing and ion release, while Au is more chemically stable and biocompatible, but more expensive and sometimes less field-enhancing than Ag [138].

Biocompatibility and environmental safety are equally important. Leaching of nanoparticles, ion release, toxic residues from synthesis, and persistent nanomaterials can pose health or ecological risks. This is particularly relevant for in vivo diagnostics, food and water monitoring, disposable sensors, and environmental remediation devices. Even Au nanostructures require careful surface functionalization to prevent aggregation, protein corona formation, immune response, or nonspecific adsorption in biological fluids [139].

Protective coating provides a practical solution. Silica shells, gold shells, polymer coatings, oxide barriers, and core–shell designs can improve colloidal stability, prevent oxidation, reduce ion release, and preserve plasmonic activity [140]. Alloying, encapsulation, and alternative materials such as doped semiconductors, nitrides, and two-dimensional materials may also improve durability in harsh environments [141]. Mechanical stability is another concern, especially for flexible, wearable, paper-based, or portable sensors, where bending, delamination, cracking, or abrasion can alter optical responses [142].

Future systems should integrate stability and safety from the beginning of material design. The most promising direction is to use protected plasmonic cores, inert or biodegradable coatings, low-toxicity synthesis routes, and robust substrate architectures. Stability testing should include repeated sensing/regeneration cycles, long-term storage, temperature variation, humidity, salinity, biological fluids, and real environmental matrices. Without such validation, high sensitivity alone will not be sufficient for practical translation.

### 6.3. Fabrication and Scalability

Fabrication scalability remains a major barrier to commercialization. Many high-performance plasmonic sensors depend on precisely engineered nanostructures, including nanogaps, nanodisks, nanohole arrays, nanoparticle clusters, metasurfaces, and SERS hot-spot substrates [143]. These architectures often require electron-beam lithography, focused-ion-beam milling, or other expensive low-throughput methods. Although these techniques provide excellent structural control, they are not ideal for producing large-area, low-cost, disposable, or mass-manufactured sensors [144].

Reproducibility is especially difficult for hotspot-based sensors. Small variations in particle size, gap distance, roughness, coating thickness, or array periodicity can produce large changes in field enhancement and signal intensity [145]. This variation limits quantitative accuracy and makes device-to-device comparison difficult. Random nanoparticle assemblies are inexpensive and scalable, but their optical response is often less uniform. Highly ordered lithographic structures are more reproducible, but cost and throughput remain limiting factors.

Therefore, scalable fabrication strategies are essential. Nanoimprint lithography, soft lithography, nanosphere lithography, anodic aluminum oxide templates, laser interference lithography, roll-to-roll printing, inkjet printing, dip-coating, and self-assembly offer promising routes for larger-area production [146]. Hybrid fabrication strategies may be most practical: top-down methods can define device architecture, while bottom-up synthesis can provide plasmonic nanoparticles with controlled shape and composition. However, successful commercialization also requires integration with microfluidics, optics, packaging, electronics, and data processing modules.

Future fabrication should prioritize reproducibility, process simplicity, material efficiency, and compatibility with disposable or flexible formats. Instead of pursuing only maximum enhancement factors, plasmonic substrates should be optimized for batch uniformity, calibration stability, manufacturability, and acceptable cost per test. Scalable plasmonic sensing will require not only advances in nanofabrication but also quality-control metrics that define acceptable variation across chips, batches, and field devices.

### 6.4. Standardization and Regulatory Issues

Standardization is essential to translate laboratory innovations into practical applications. Although many plasmonic sensors report excellent sensitivity, differences in substrate preparation, functionalization, measurement conditions, calibration, and data analysis make cross-study comparison difficult. Future studies should, at a minimum, report the substrate-fabrication route, structural reproducibility metrics, surface functionalization protocol, storage conditions, calibration model, number of independent chips or batches tested, matrix composition, interference-study design, real-sample recovery, reference-method comparison, and regeneration or lifetime data, when applicable. SERS studies should include spatial variance and map-based reproducibility alongside enhancement claims, while SPR and LSPR studies should consistently report baseline drift, temperature sensitivity, linewidth, and reference channel strategy.

Quantitative sensing requires reliable calibration. Benchmark plasmonic devices against certified standards, reference materials, and established analytical methods such as chromatography, mass spectrometry, electrochemical assays, or validated immunoassays. Calibration curves should cover realistic concentration ranges and account for interfering species, pH variation, ionic strength, temperature changes, and matrix effects. For SERS substrates, standardized reporting of enhancement factors and spatial mapping is also needed. For SPR and LSPR sensors, report resonance shift, linewidth, drift, response time, regeneration, and lifetime consistently.

Regulatory acceptance requires proof of sensitivity, specificity, reproducibility, robustness, safety, and stability under intended use conditions [147]. Environmental agencies, clinical authorities, and industrial users will require validation data that show that plasmonic sensors perform reliably over time, across devices, and in real samples. Safety documentation is also necessary, particularly when sensors contain nanomaterials that may encounter water, food, biological samples, or ecosystems. Commercial devices will require manufacturing quality systems, batch control, and traceability.

Future progress should include interlaboratory validation, blind testing, standard reference substrates, shared datasets, and harmonized reporting guidelines. Collaboration among researchers, manufacturers, standards organizations, and regulatory bodies will be essential. The long-term success of plasmonic adsorption technologies will depend on demonstrating that they are not only sensitive and innovative but also reproducible, safe, traceable, and reliable enough to replace or complement established analytical methods. Without standardized protocols, it is challenging to determine whether a sensor’s performance is truly superior or merely measured under more favorable conditions.

Overall, the major limitation of plasmonic adsorption sensing is the gap between ideal laboratory performance and robust field operation. Future research should focus on SNR optimization, antifouling and selective interfaces, durable and biocompatible materials, scalable manufacturing, standardized validation, and regulatory readiness. Addressing these challenges will determine whether plasmonic adsorption-based platforms can become trusted tools for environmental monitoring, biomedical diagnostics, food safety, and industrial sensing.

## 7. Future Roadmap

The future of plasmonics-enhanced adsorption technologies will depend on moving beyond isolated sensor demonstrations to intelligent, multifunctional, sustainable, and commercially viable platforms. The main direction is clear: plasmonic systems must combine selective adsorption interfaces, robust nanostructures, portable readout, data-driven interpretation, scalable fabrication, and regulatory readiness. Five interconnected priorities will shape future progress: artificial intelligence, new spectral and quantum regimes, hybrid multifunctionality, green materials, and commercialization.

### 7.1. Integration with AI and Machine Learning

Artificial intelligence (AI) and ML will become central tools for both plasmonic sensor design and signal interpretation [113]. Plasmonic adsorption sensors often generate complex optical data, including SERS spectra, resonance shifts, image patterns, time-dependent kinetic traces, and multiplexed array responses. Conventional analysis based on single-peak shifts or intensity thresholds may miss weak signals or fail in complex matrices. ML can use the full information content of these data to distinguish target responses from noise, drift, overlapping peaks, and nonspecific adsorption [148].

AI is especially valuable for SERS and multiplexed LSPR arrays, where the analyte fingerprints may overlap with background signals. Supervised models can classify and quantify pollutants, biomarkers, or gas mixtures from spectral patterns, while unsupervised methods can reveal hidden clusters, matrix effects, or abnormal sensor behavior. AI can also support real-time baseline correction, denoising, feature extraction, and decision-making on portable devices. In future field systems, AI should not be limited to post-analysis; it should actively guide sensing operation by optimizing measurement conditions, selecting wavelength windows, identifying drift, and triggering recalibration.

AI-assisted design is equally important. Deep learning, inverse design, and evolutionary algorithms can accelerate the discovery of nanostructures with targeted resonance wavelengths, stronger hot spots, narrower linewidths, or improved adsorption sensitivity. This approach can reduce trial-and-error fabrication and reveal geometries that are difficult to identify using conventional intuition.

Supplementary Figure S9 highlights the role of AI/ML in plasmonic adsorption-based sensing, including spectral analysis, noise reduction, nanostructure optimization, and autonomous decision-making. Future progress should prioritize interpretability, since environmental and clinical applications require explainable, validated, and reproducible models supported by standardized datasets, uncertainty estimation, and field validation.

### 7.2. Near-Infrared (NIR) II and Quantum Plasmonics

Expanding plasmonic sensing to the second near-infrared window (NIR-II, approximately 1000–1700 nm) and quantum-enhanced regimes will extend both sensitivity and application scope. NIR-II operation is attractive because it offers deeper biological penetration, less scattering, reduced autofluorescence, and compatibility with fiber optic and telecommunication platforms [149]. For environmental monitoring, NIR-II plasmonic sensors could enable remote or distributed readout through optical fibers, supporting applications such as pipeline monitoring, water network surveillance, and field-based sensing arrays. Achieving a strong NIR-II plasmonic response requires careful material and geometry design. Au nanorods, nanoshells, nanowires, and coupled nanostructures can shift LSPR peaks to longer wavelengths, while doped semiconductors, copper chalcogenides, transparent conducting oxides, and other infrared plasmonic materials provide additional design flexibility [150]. Future NIR II plasmonics should focus on low-loss materials, stable coatings, water-compatible operation, and portable NIR detection hardware.

Quantum plasmonics offers another route to surpass the limits of classical sensing. Quantum light sources, entangled photons, squeezed light, and quantum-enhanced detection can reduce measurement noise and potentially improve refractive-index or molecular sensing beyond the classical shot-noise limit [151]. At the nanoscale, quantum tunneling, charge transfer, and nonlocal effects become important in subnanometer gaps, where classical electromagnetic models no longer fully describe plasmonic behavior [152]. These effects may enable new sensing modalities based on quantum conductance, strong coupling, or single-molecule interactions.

The future impact of NIR-II and quantum plasmonics will depend on whether these concepts can be translated into stable, affordable, and reproducible devices. Near-term progress is likely to come from NIR-II fiber-compatible sensors and low-noise optical readout. Quantum-enhanced plasmonic sensing remains more fundamental, but it could ultimately enable ultralow-noise detection, single-molecule analysis, and new molecular identification strategies.

### 7.3. Multifunctional and Hybrid Systems

Future plasmonic adsorption platforms will increasingly combine sensing with additional functions, including imaging, therapy, photocatalysis, sterilization, remediation, and energy conversion. This multifunctional direction is especially important because plasmonic nanostructures can concentrate light, generate heat, produce hot carriers, enhance Raman or fluorescence signals, and interact strongly with adsorbed molecules on the same interface [153,154,155,156].

In biomedical applications, plasmonic nanoparticles can support theranostics by combining diagnosis and treatment. Au nanorods, nanoshells, nanostars, and hybrid nanostructures can detect biomarkers through LSPR or SERS and then generate photothermal heat for tumor ablation or antimicrobial treatment [153,154]. Plasmonic systems can also enhance photodynamic therapy by increasing local excitation of photosensitizers and improving reactive oxygen species generation. In these platforms, adsorption and targeting determine where the plasmonic material accumulates, while optical excitation determines the detection or treatment outcome.

Hybrid magneto-plasmonic, semiconductor–plasmonic, and polymer–plasmonic systems further expand functionality. Magnetic components enable separation, guidance, and magnetic resonance contrast; semiconductors enable photocatalytic reactions; polymers and hydrogels provide molecular recognition, drug loading, or environmental responsiveness. In environmental technology, multifunctional systems may detect pollutants and simultaneously degrade them through plasmon-enhanced photocatalysis or photothermal treatment.

Information-technology applications such as heat-assisted magnetic recording (HAMR) and plasmonic computing rely on field localization and nanoscale energy confinement rather than molecular adsorption in the strict interfacial-chemistry sense used throughout this review. We therefore discuss them only briefly as related plasmonic phenomena, not as core adsorption-based sensing examples [157].

The main challenge is functional balance. Adding multiple components can reduce plasmonic enhancement, block adsorption sites, increase toxicity, or complicate fabrication. Therefore, future hybrid platforms should follow a clear functional hierarchy: capture, transduction, action, and regeneration. The most promising systems will integrate sensing and response without sacrificing selectivity, reproducibility, or safety.

### 7.4. Lifecycle and Deployment Considerations

For disposable environmental sensors, material consumption, metal release, and waste handling affect deployment decisions. Au, Ag, Al, Cu and alternative substrates should be compared at the intended lifetime and scale, while maintaining the recognition chemistry and adsorption access needed for reliable measurement [158,159].

**Figure 7:**
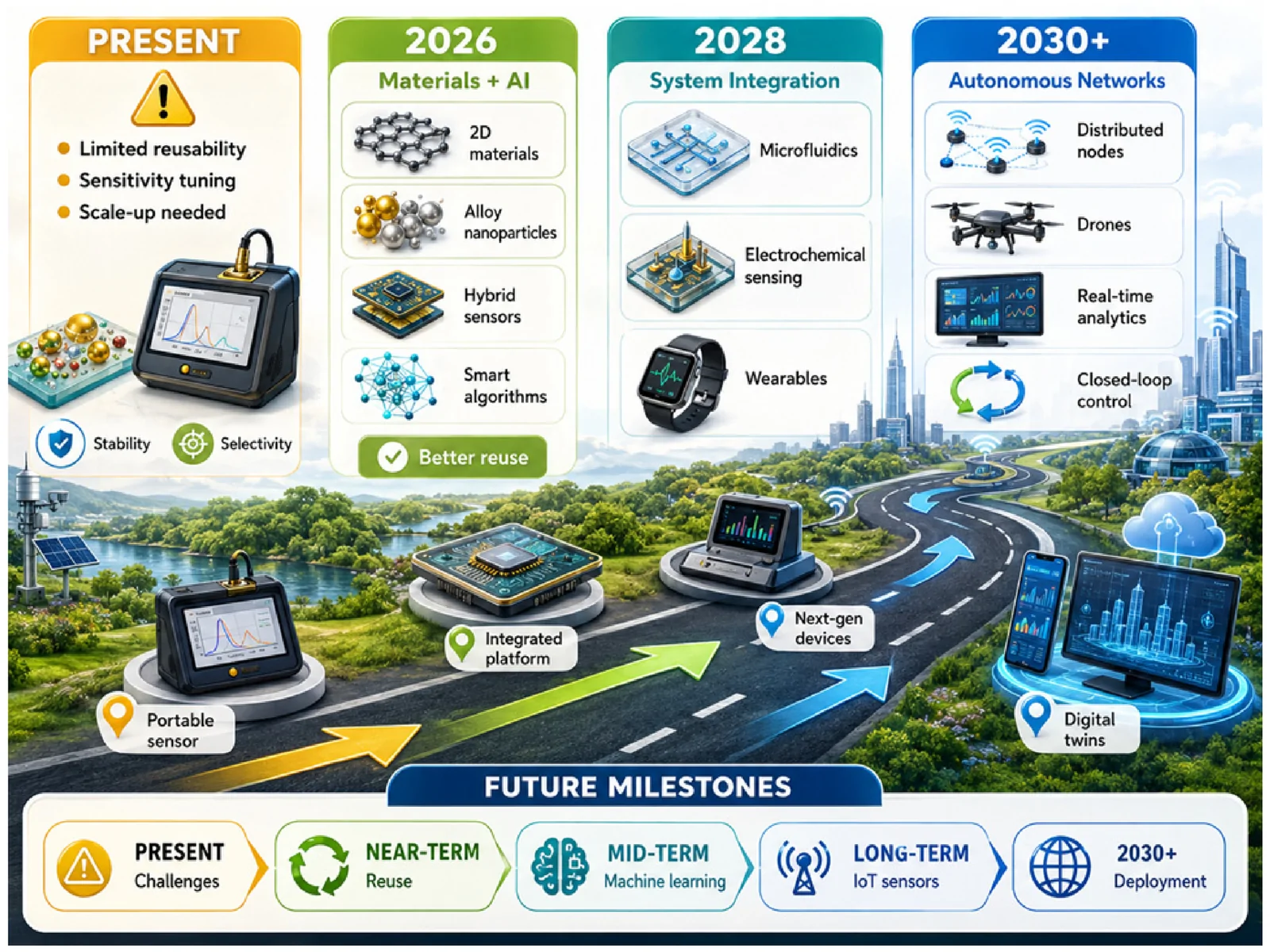
Roadmap for plasmonics-enhanced adsorption technologies. Current challenges (reusability, sensitivity, scalability) have been short-term (MXenes, nitrides, alloys), mid-term (microfluidics, wearables, ML), and long-term (autonomous IoT networks, real-time analytics, commercial devices) advances.

Figure 7 is a conceptual sequence of dependencies rather than a forecast that a particular material or device will be ready in the stated year. Improved alloys, two-dimensional layers, and protective coatings matter only if they maintain selective adsorption and near-field access over repeated cycles and storage. Microfluidics can control residence time and remove particles but also introduces recovery losses and fouling; connected readers can compensate for drift only when trained and checked on representative matrices. Distributed monitoring further requires batch-to-batch calibration, reference-method comparison, maintenance procedures, and traceable decisions when a threshold is exceeded. Thus, the transition from a portable prototype to an autonomous network is governed as much by validated measurement uncertainty and interface lifetime as by materials development or wireless connectivity. Interpret the milestones in the figure as research priorities, not demonstrated performance or guaranteed dates.

### 7.5. Towards Commercialization

Commercialization is the final and most demanding step in the roadmap. A plasmonic adsorption sensor must be more than sensitive; it must be manufacturable, affordable, stable, calibrated, user-friendly, and validated under real operating conditions. The path begins with robust prototypes, followed by field validation, scalable manufacturing, cost reduction, standardization, regulatory approval, and market adoption.

Prototyping should focus on complete systems rather than isolated sensing chips. Practical devices must include sample handling, adsorption interfaces, optical readout, calibration, data analysis, and packaging. Field testing is essential to evaluate drift, fouling, temperature effects, humidity, mechanical durability, response time, and regeneration. Only sensors that perform reliably in real samples can move toward deployment.

Manufacturing and cost remain decisive. Scalable production methods such as roll-to-roll processing, nanoimprint lithography, printable plasmonics, colloidal assembly, and semiconductor-compatible fabrication should be prioritized. Disposable components should be low-cost, while reusable readers should integrate stable optics, reference channels, automated calibration, and wireless data transfer. Commercial success will depend on finding the right balance between disposable sensor cartridges and reusable electronic/optical units.

Standardization and regulatory validation must be addressed early. Sensors should be benchmarked against established analytical methods, tested with certified standards, and validated on realistic matrices. For environmental applications, acceptance will require reproducible data across batches, laboratories, and field sites. For biomedical or food-related applications, safety, biocompatibility, and quality control will be even more stringent.

User-oriented design is equally important. Commercial devices should provide clear results rather than raw spectra, with software that reports concentration, uncertainty, safety threshold, and recommended action. Integration with smartphones, cloud databases, and Internet of Things (IoT) networks can support large-scale environmental monitoring. AI-driven analysis can also enable automated interpretation, predictive alerts, and adaptive calibration.

Supplementary Figure S10 summarizes the commercialization roadmap from laboratory research to field deployment, covering prototyping, scalable manufacturing, cost reduction, standardization, regulatory approval, user-centered design, and IoT/AI integration. It highlights that successful translation requires coordinated advances in science, engineering, manufacturing, data systems, and regulation.

## 8. Conclusions and Prospects

A central conclusion of this review is that adsorption is not an auxiliary event on top of plasmonic transduction, but an interfacial process that largely determines whether plasmonic sensing remains a laboratory demonstration or becomes a reliable analytical technology. Therefore, the most important design variables extend beyond resonance position, field enhancement, and hot-spot density. They also include surface chemistry, binding site accessibility, adsorption kinetics, fouling resistance, matrix tolerance, regeneration, and reproducibility across devices and batches.

Accordingly, future progress should be evaluated using application-relevant criteria rather than idealized optical performance alone. For environmental deployment, the key benchmarks are selectivity in complex matrices, robustness under realistic ionic strength and fouling conditions, analytical validation against established reference methods, scalable fabrication, and transparent reporting of uncertainty and reproducibility.

Significant progress is already being made in addressing these limitations. Advanced surface chemistries and molecularly imprinted polymers improve selectivity; protective coatings, hybrid materials, and sustainable plasmonic platforms enhance stability and biocompatibility; scalable fabrication methods enable large-area, low-cost production; and standardization efforts are improving reproducibility and regulatory readiness. At the same time, artificial intelligence and machine learning are expected to strengthen spectral interpretation, noise suppression, pattern recognition, sensor calibration, and inverse nanostructure design.

The next generation of plasmonic adsorption systems is likely to be intelligent, portable, multifunctional, and sustainable. Future platforms may integrate SPR, LSPR, or SERS sensing with microfluidics, smartphone readout, cloud connectivity, and Internet of Things monitoring networks. Emerging directions such as NIR-II plasmonics, quantum-enhanced sensing, hybrid theranostic systems, and plasmonic photocatalytic remediation will further expand the role of plasmonics beyond detection towards real-time intervention. In environmental applications, this may lead to detect-and-remediate systems that identify pollutants and initiate light-driven degradation or purification.

Sustainability will also be essential. Sensors intended to protect environmental and human health should minimize their own ecological footprint by using earth-abundant materials, green synthesis, recyclable components, biodegradable substrates, and safe end-of-life disposal strategies.

Overall, plasmonics-enhanced adsorption is moving from proof-of-concept sensing to practical analytical platforms. Its broad impact will depend on integrating nanophotonic design, adsorption chemistry, materials science, scalable manufacturing, data analytics, and regulatory validation. With continued multidisciplinary progress, plasmonic adsorption-based technologies are well positioned to become important tools for real-time environmental monitoring, food safety, biomedical diagnostics, industrial sensing, and sustainable remediation. Their greatest promise lies not only in the detection of trace analytes with high sensitivity, but also in enabling faster decisions, earlier warnings, and more effective protection of public health and the environment.

## Figures and Tables

**Table 1:** Plasmonic adsorption-based environmental sensing platforms.

Environmental Matrix	Target Contaminants	Plasmonic Strategy	Recognition Interface	Typical Performance	Refs.
Water	Heavy metals (Pb^2+^, Cd^2+^, Cr^6+^, Hg^2+^)	LSPR, colorimetric	Chelating ligands, DNAzymes, thiols	nM–ppb range; rapid screening	[42]
	Organic pesticides, antibiotics	SERS, LSPR	MIPs, aptamers, hydrophobic surfaces	nM–µM range; molecular fingerprinting	[43,44]
	Microplastics	SERS, LSPR	Polymer-specific adsorption	µm–sub-µm particles	[45,46]
Air	NO_2_, NH_3_, VOCs	LSPR, SPR	MOFs, metal oxides, porous coatings	ppm–sub-ppm range; preconcentration	[47,48]
Soil/Food	Pesticides, pathogens, toxins	SERS, LSPR	Antibodies, MIPs, aptamers	ppb–ppm range; portable screening	[49]

## Data Availability

No new data were created or analyzed in this study. Data sharing is not applicable to this article.

## Supplementary Materials

The following supporting information can be downloaded at https://www.mdpi.com/article/10.3390/mi17101168/s1. See the Supplementary Material for four tables and ten figures providing additional support for the adsorption-governed plasmonic sensing framework. Tables S1–S4 summarize the sensing modalities and adsorption mechanisms, the influence of interfacial interactions on SPR/LSPR/SERS signals, emerging plasmonic materials and fabrication techniques, and expanded environmental sensing examples with representative detection limits and references. Figures S1–S10 illustrate the adsorption-controlled sensing workflow, LSPR near-field enhancement, multiscale modeling, representative sensor architectures, adsorption isotherm and kinetic models, field sampling strategies, broader plasmonic applications, environmental remediation concepts, AI/ML integration, and the commercialization roadmap from laboratory validation to field deployment [7,43,45,160,161,162,163,164,165,166,167,168,169,170].
