# Supplementary material for: Adsorption-Governed Plasmonic Sensing for Environmental Management and Monitoring Programs
Source: Micromachines (Basel). 2026 Oct 7;17(10):1168. doi: 10.3390/mi17101168 (PMC13707909; doi:10.3390/mi17101168)
Supplement: Supplementary file 1 [file micromachines-17-01168-s001.zip › micromachines-4586853-supplementary.pdf]

Supplementary material

Adsorption-Governed Plasmonic Sensing for Environmental Management and Monitoring Programs

Yuan-Fong Chou Chau[1,2*], Abdul Hanif Mahadi[1,3], Roshan Thotagamuge[4], Muhammad Raziq Rahimi Kooh[1,3*]

1 Centre for Advanced Material and Energy Sciences, Universiti Brunei Darussalam, Tungku Link,
Gadong BE1410, Brunei; hanif.mahadi@ubd.edu.bn
2 Applied Physics, Faculty of Science, Universiti Brunei Darussalam, Tungku Link, Gadong BE1410, Brunei
3 Chemical Sciences, Faculty of Science, Universiti Brunei Darussalam, Tungku Link, Gadong BE1410, Brunei
4 Department of Optometry, Faculty of Allied Health Sciences, University of Sri Jayewardenepura,
Nugegoda 10250, Sri Lanka; roshan@sjp.ac.lk
* Correspondence: chou.fong@ubd.edu.bn (Y.-F.C.C.); raziq.kooh@ubd.edu.bn (M.R.R.K.)

Supplementary

Table S1 further summarizes representative plasmonic nanostructures used to enhance adsorption, including colloidal nanoparticles, nanorods, nanocubes, nanoplates, core–shell structures, nanoporous substrates, ordered arrays, and hybrid composites with polymers, metal–organic frameworks, semiconductors, or two-dimensional materials. These structures highlight a central materials-design opportunity: adsorption performance can be tuned through morphology, surface area, hot-spot density, chemical functionality, and hybrid interfacial architecture.

Table S1. Plasmonic sensing modalities and adsorption mechanisms

Sensing Modality	Colloidal NPs	Nanorods	Core-Shell	Nanoporous	Ordered Arrays	Hybrid Composites
SPR	✓	✓	✓	✓	✓	✓
LSPR	✓	✓	✓	✓	✓	✓
SERS	✓	✓	✓	✓	✓	✓
Key tunable parameters	Morphology, surface area, hot-spot density, interfacial chemistry	Aspect ratio, resonance tuning	Shell thickness, protection	Pore size, surface area	Periodicity, collective modes	Synergistic adsorption, selectivity

Table S2 summarizes the major adsorption mechanisms relevant to SPR, LSPR, and SERS, including physisorption, chemisorption, electrostatic and ion-exchange adsorption, charge-transfer interactions, competitive adsorption and fouling, and speciation-dependent binding, together with their effects on plasmonic signals and environmental sensing performance.

Table S2 – Adsorption Mechanisms and Their Impact on Plasmonic Signals

Adsorption mechanism	Description of interactions	Impact on plasmonic signals (SPR, LSPR, SERS)	Environmental & sensing implications
Physisorption (van der Waals/hydrophobic)	Weak, nonspecific adsorption driven by van der Waals forces, hydrogen bonding, or hydrophobic interactions; typically reversible.	Primarily adds mass and changes the effective refractive index in the evanescent field; produces modest resonance shifts without significantly altering plasmon damping.	Suitable for reversible sensing, but low specificity makes physisorption susceptible to matrix interference and baseline drift.

Chemisorption (covalent/coordinate)	Strong adsorption via covalent or coordinate bonds (e.g., thiol-metal or ligand exchange); often irreversible.	Modifies the interfacial electronic structure and charge distribution, inducing pronounced resonance shifts or damping, and sometimes introducing new spectral features.	Enables selective sensing when functional groups are employed, but may reduce quality factors and requires surface regeneration for reuse.
Electrostatic and ion-exchange adsorption	Coulombic attraction and ionexchange between charged analytes and functionalized surfaces; includes protonation/deprotonation processes.	Alters local refractive index and near-field distribution; sensitivity depends on Debye length and ionic strength; can broaden spectral lines.	Highly pH and ionic-strength dependent; important for detecting charged pollutants (e.g., metal ions), but subject to screening and competitive binding.

Charge-transfer and dipole interactions	Adsorbate–metal charge transfer and induced dipole formation that modify the electronic density.	Influences both dielectric function and damping; can enhance SERS via chemical enhancement pathways or quench plasmon resonances; often shifts resonance frequencies.	Crucial for molecular fingerprinting by SERS; facilitates attomolar detection but complicates quantitative SPR/LSPR calibration.
Competitive adsorption and fouling	Multiple species compete for binding sites or form fouling layers (e.g., NOM, biofilms) blocking active areas.	Reduces available sites, increases baseline noise, and decreases sensitivity; may cause irreversible changes to the sensor response.	Common in environmental samples; underscores the need for pretreatment and anti-fouling coatings and dynamic adsorption models.

Speciation-dependent binding	Adsorption strength depends on the speciation (e.g., oxidation state), pH, or ionic environment.	Causes variable resonance shifts and response factors, influences binding kinetics and can change the sign of the signal.	Calibration must account for environmental conditions; exploited for speciation analysis but introduces complexity in quantitative sensing.

Table S3 lists representative emerging plasmonic materials and associated fabrication strategies relevant to adsorption-controlled sensing. For each category, we list typical fabrication techniques and key advantages, highlighting tunable resonance wavelengths, high thermal stability, low losses, and compatibility with scalable or flexible platforms. These emerging materials and fabrication strategies expand the design space for future plasmonic sensors and devices.

Table S3. Emerging plasmonic materials and fabrication techniques.

Emerging Plasmonic Materials	Key Properties	Typical Fabrication Techniques	Description/Advantages
Refractory metal nitrides (e.g., TiN, ZrN)	High melting points (>2,500 °C), CMOS compatible, tunable plasmonic resonances extending into the near-IR	Reactive magnetron sputtering, atomic layer deposition (ALD), pulsed laser deposition	Alternatives to noble metals for high-temperature or harsh environments; suitable for integrated photonics and thermoplasmonic applications.

Doped transparent oxides (e.g., aluminum-doped zinc oxide, indium tin oxide)	Low intrinsic losses in the near-IR, tunable carrier density via doping, and compatibility with flexible substrates	Sol-gel deposition, radiofrequency sputtering, nanoprinting	Offer tunable localized surface plasmon resonances (LSPRs) in the mid-IR; attractive for sensing and smart window applications where transparency is required.
Topological insulators (e.g., Bi_2Se_3, Sb_2Te_3)	Support Dirac surface states with low dissipation; host surface plasmons protected against back-scattering	Molecular beam epitaxy (MBE), solution-phase synthesis	Provide robust plasmonic modes that are less sensitive to defects; useful for quantum plasmonics and ultrafast photonics.
2D materials (graphene, MXenes, transition-metal dichalcogenides)	Atomically thin, highly tunable carrier concentration, strong light–matter interaction	Chemical vapor deposition (CVD), mechanical exfoliation, selective etching	Enable broadband, dynamically tunable plasmonic responses; can be combined with metal nanoparticles to form hybrid structures with enhanced sensing performance.
Hybrid metal–dielectric metasurfaces	Combine high-index dielectrics (Si, GaN) with metals to engineer magnetic and electric resonances with reduced losses	Ebeam lithography, nanoimprint lithography, self-assembly of nanoparticles on patterned templates	Allows tailoring of dispersion for achromatic and multifunctional metasurface metalenses, with improved field confinement and efficiency compared to all-metal structures.

Flexible plasmonic substrates (polymer–metal nanocomposites)	Mechanical Flexibility and stretchability; maintain plasmonic properties upon deformation	Roll-to-roll nanoimprinting, transfer printing of nanostructures, inkjet printing of metal nanoparticle inks	Facilitate the integration of plasmonic sensors into wearables and curved surfaces; scalable, cost-effective fabrication suitable for environmental monitoring in the field.

Table S4 provides expanded examples of plasmonic adsorption-based environmental sensing platforms across water, soil, food, air, and point-of-use applications. It summarizes representative target analytes, plasmonic detection strategies, recognition or adsorption interfaces, signal readout modes, reported detection-limit ranges, and DOI-linked references. This table provides detailed performance benchmarks and application-specific examples for comparison.

Table S4. Expanded examples of plasmonic adsorption-based environmental sensing platforms with representative detection limits and references.

Environmental matrix	Target analyte(s)	Plasmonic platform	Recognition/adsorption interface	Signal readout	Reported or typical detection limit	Key remarks	Ref.
Drinking water / tap water	Hg^{2+}	Functionalized Au nanoparticles	Thiol-, amine-, DNA-, or ligand-functionalized AuNPs	Colorimetric aggregation or LSPR shift	Low nM to ppb level	Simple visual or smartphone-readable method; suitable for rapid screening but vulnerable to ionic-strength effects	[1]
Drinking water / groundwater	Hg^{2+}	AuNP-enhanced fiber-optic SPR sensor	Sulfur-modified Au film and AuNP	SPR wavelength shift	~8.15 nM	Strong Au–S–Hg^{2+} interaction improves selectivity;	[2]

			coupling layer			suitable for real-water validation	
Drinking water / aqueous samples	Hg^{2+}	Bifunctionalized Ag nanoparticles	Bifunctional ligands on AgNP surface	LSPR/colorimetric response	Low nM range	High sensitivity for Hg^{2+}; Ag oxidation and long-term stability require attention	[3]
Water/buffer / environmental extracts	Pb^{2+}	DNAzyme- or aptamer-linked AuNPs	Metal-ion-responsive DNAzyme/aptamer interface	Aggregation-induced color or LSPR shift	nM to low ppb range	High selectivity can be achieved using ion-specific nucleic-acid receptors; matrix calibration is necessary	[4]
Water/wastewater	Organic dyes, e.g., Rhodamine 6G, methylene blue	Ag or Au SERS substrates	Direct adsorption on plasmonic hot spots	SERS fingerprints	10^{-9}–10^{-12} M for model dyes	Very high sensitivity in clean systems; quantitative use requires uniform hot spots and antifouling protection	[5]
Wastewater/river water	Polycyclic aromatic hydrocarbons (PAHs)	AuNP–MIP hybrid SERS sensor	Molecularly imprinted polymer film on AuNP assemblies	SERS fingerprint after selective adsorption	Low nM level	MIP traps PAHs close to plasmonic hot spots, improving selectivity in complex samples	[6]
River water /	Pesticides and herbicides	MIP–plasmonic SERS or	Molecularly imprinted polymer or	SERS peaks or LSPR shift	nM to sub-nM range,	Useful for selective pesticide	[7]

agricultural runoff		LSPR sensor	selective polymer coating		depending on analyte	capture; polymer thickness must be optimized to avoid reducing near-field coupling	
Water / fruit-surface extracts	Organophosphate pesticides	Filter-paper AgNP SERS substrate	Hydrophobic paper surface concentrating pesticide residues	SERS fingerprint	Sub-nM level	Low-cost, flexible, and field-friendly; sample extraction or wiping step usually required	[7]
Water/beverage samples	Pesticide residues	Extraction-integrated plasmonic SERS platform	Floating or porous plasmonic substrate for enrichment	SERS fingerprint	nM to low ppb range	Combines preconcentration with SERS detection; useful for complex liquid samples	[8]
Marine water / surface water	Microplastics and nanoplastics	SERS-active nanopillar arrays or plasmonic nanoprobes	Trapping or adsorption of polymer fragments near hot spots	Polymer-specific SERS spectra	Typically µm to sub-µm particles; nanoscale detection reported in optimized systems	Provides chemical identification of polymer type; sample filtration/preconcentration is important	[9]
Soil extract	Pb^{2+} and other	AuNP colorimetric or	DNAzyme, thiol, or chelator-	Colorimetric aggregatio	Low ppb to sub-	Requires extraction from soil before	[4]

	heavy metals	LSPR assay	functionalized nanoparticles	n or LSPR shift	ppm range in extracts	sensing; suitable for field screening rather than definitive regulatory quantification	
Soil extract / contaminated soil	PAHs and persistent organic pollutants	MIP–SERS plasmonic substrate	Imprinted polymer layer for PAH capture	SERS fingerprint	Low nM range	Selective adsorption improves discrimination from humic substances and other soil organics	[6]
Food surface / produce	Pesticide residues, e.g., thiram, parathion, malathion	Paper-based or swab-based SERS strip	AgNP/AuNP-loaded paper, hydrophobic coating, or adhesive nanomesh	SERS fingerprint using portable Raman reader	ng cm^{-2} to sub-nM equivalent concentration	Promising for rapid on-site screening; surface recovery efficiency must be validated	[7]
Food extracts / grains / milk	Mycotoxins, antibiotics, hormones	SPR/LSPR immunosensor or MIP–plasmonic sensor	Antibodies, aptamers, or MIP recognition layer	SPR angle shift, LSPR shift, or SERS signal	pg mL^{-1} to ppb range, depending on receptor and matrix	High selectivity possible, but matrix pretreatment and receptor stability are critical	[10]
Indoor air / atmospheric air	VOCs, e.g., ethanol, methanol, acetone, toluene	LSPR optical fiber sensor	AuNP-coated fiber tip decorated with MOF layer	LSPR intensity or wavelength change	ppm to sub-ppm range	MOF layer enriches VOCs and improves selectivity; humidity correction is needed	[11]

Indoor / industrial air	NO_2	Au/ZnO or metal–oxide/plasmonic thin film	Chemoselective oxide layer or ligand-modified plasmonic surface	SPR/LSPR shift or extinction change	ppb to low ppm range	Fast response possible at ambient conditions; cross-sensitivity to humidity and oxidizing gases must be controlled	[12]
Industrial / urban air	CO_2, CH_4, NH_3	Plasmonic metasurface or perfect absorber	Polymer, MOF, or porous adsorption layer tuned to target gas	Resonance intensity modulation or IR-coupled absorption	ppm to sub-ppm range for optimized devices	Compact spectrometer-free gas sensing possible when plasmonic resonance overlaps molecular absorption band	[13]
Air / vapor-phase security monitoring	Nitroaromatic vapors and hazardous VOCs	SERS-active Ag/Au substrate	Polymer capture coating or porous hot-spot substrate	SERS vapor fingerprint	sub-ppm to ppb range in optimized systems	Useful for vapor identification; adsorption enrichment is essential due to low gas-phase molecular density	[7]
Point-of-use water testing	Heavy metals, pH, colorimetric pollutants	Smartphone-read AuNP or AgNP plasmonic assay	Ligand-functionalized nanoparticles on paper or cartridge	RGB/color intensity or LSPR shift	ppb to low ppm range, depending on analyte	Low-cost and accessible; camera calibration, lighting control, and app-based correction are required	[14]

Portable environmental monitoring	Pesticides, toxins, heavy metals, pathogens	Disposable plasmonic chip or paper strip with handheld reader	Aptamer, antibody, MIP, or ligand-functionalized surface	SERS, LSPR, or SPR readout

Supplementary Figure S1 identifies adsorption as the central governing step in plasmonic environmental sensing. After sample collection and optional pretreatment, target analytes must selectively adsorb onto functionalized recognition layers (e.g., aptamers, molecularly imprinted polymers, thiols, or oxides) before plasmonic transduction (SPR, LSPR, or SERS) converts interfacial changes into spectral signals for detection. However, matrix interferences—natural organic matter (NOM), salts, and biofouling—directly hinder adsorption, reducing sensitivity and selectivity. The figure emphasizes that real-world performance is limited primarily by adsorption efficiency rather than optical transduction.

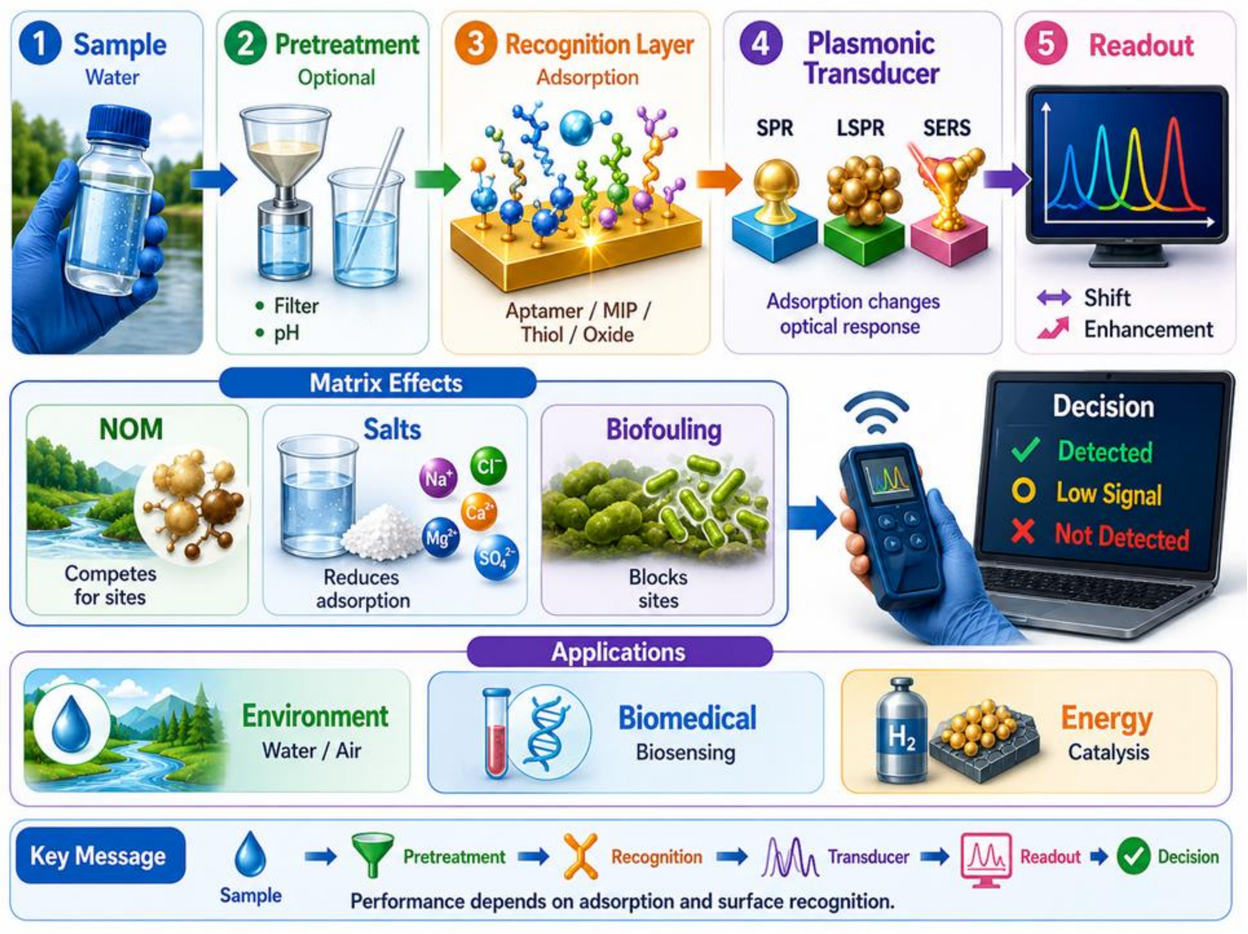

Figure S1. Adsorption-governed workflow in plasmonic sensing. From sample input to decision: pretreatment, adsorption/recognition layer formation, plasmonic signal transduction (SPR/LSPR/SERS), and spectral readout. Side arrows indicate matrix challenges (NOM, salts, biofouling) acting on the adsorption layer, showing that field performance is constrained by surface recognition, not transduction alone.

Supplementary Figure S2 shows how light excites a gold nanoparticle, creating a strong local field around the adsorbed molecule. This field enhances their Raman signal, while adsorption can shift the LSPR wavelength. The inset shows a separate, possible charge-transfer pathway that may provide additional chemical SERS enhancement when the metal and molecule are suitably coupled.

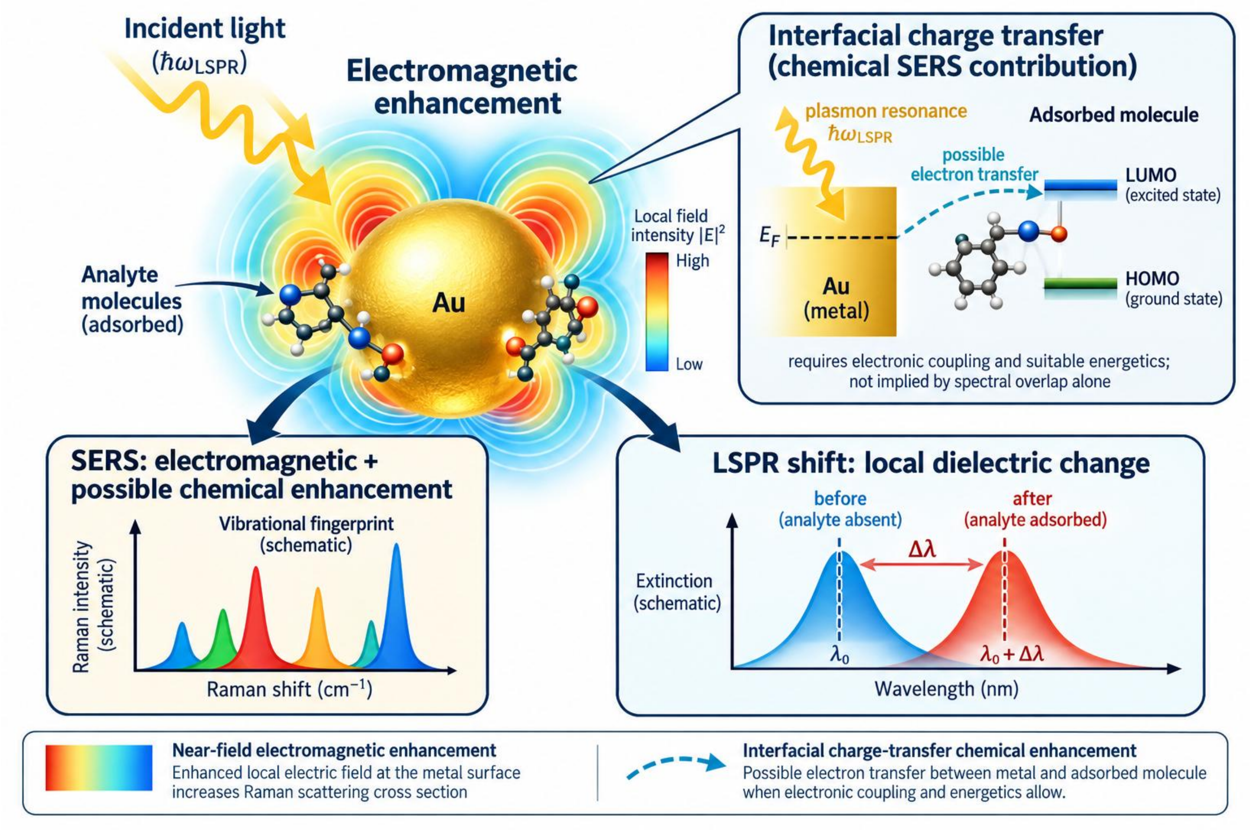

Figure S2. LSPR excitation and near-field enhancement on a noble-metal nanoparticle. Adsorption of analyte molecules amplifies SERS and induces LSPR shifts. Inset: plasmon-molecule coupling via charge transfer (chemical enhancement mechanism), not simple energy-level alignment.

Figure S3 presents a multiscale workflow linking nanostructure geometry, dielectric functions, adsorption-layer properties, and simulation methods to measurable outputs such as resonance shift, near-field distribution, and SERS enhancement. **Figure S4** compares colloidal nanoparticles, nanorod arrays, metasurface gratings, and fiber-optic probes, emphasizing that all architectures rely on the same principle: analyte adsorption modifies plasmon–matter interactions, producing detectable optical signals.

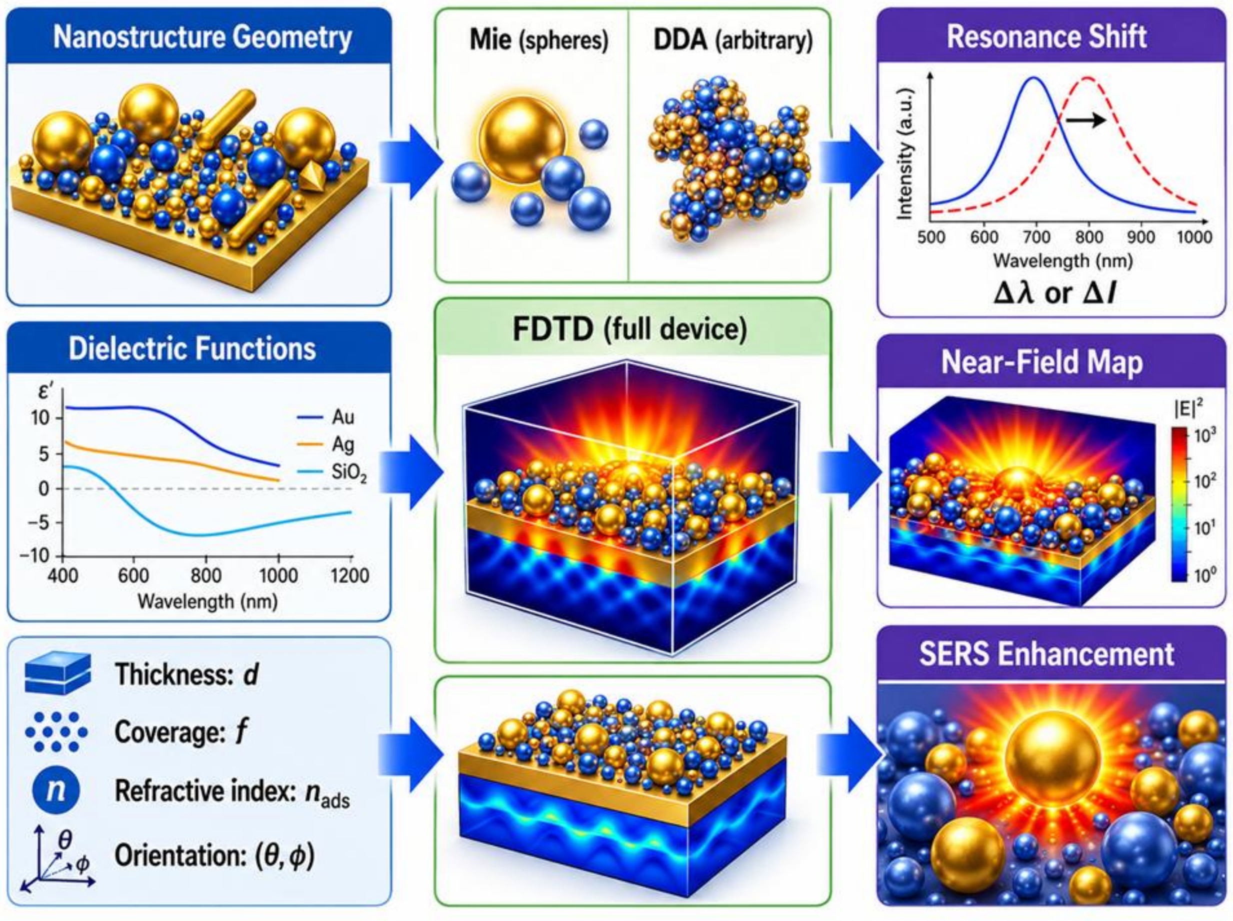

Figure S3. Modeling workflow for adsorption-governed plasmonic sensors. Inputs: nanostructure geometry, dielectric functions, and adsorption-layer properties (thickness, coverage, refractive index, orientation). Methods: Mie theory (spheres), DDA (arbitrary shapes), FDTD (full devices). Outputs: resonance shifts, near-field enhancement maps, and SERS spatial distributions.

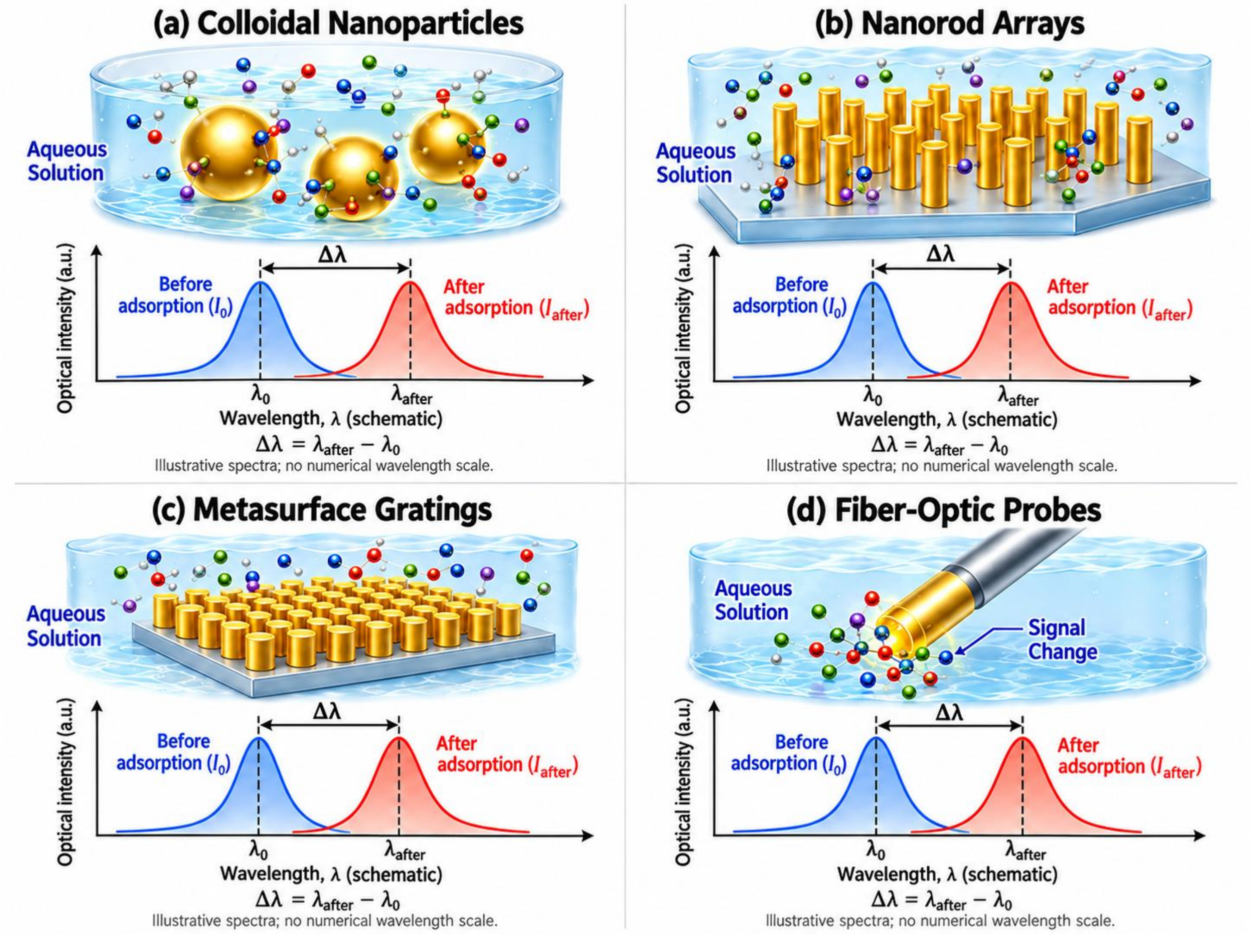

Figure S4. Plasmonic sensor architectures for adsorption-based detection: (a) colloidal nanoparticles, (b) ordered nanorod arrays, (c) metasurface gratings, and (d) fiber-optic probes. Adsorption can alter the local dielectric environment, producing spectral shifts or intensity changes. $\Delta\lambda$ denotes the difference between the peak wavelengths after and before adsorption: $\Delta\lambda = \lambda_{after} - \lambda_0$. Dashed guides mark the peak positions. Spectra are schematic and do not represent measured numerical shifts.

Figure S5 presents fundamental adsorption models for pollutant–plasmonic material interactions. The upper panels compare Langmuir and Freundlich isotherms, describing equilibrium adsorption behavior and surface heterogeneity for species such as heavy metals and volatile organic compounds (VOCs). The lower panels depict pseudo-first-order and pseudo-second-order kinetic models, illustrating time-dependent adsorption processes. Together, these models explain how adsorption capacity and kinetics govern plasmonic sensing signals, including spectral shifts and intensity variations.

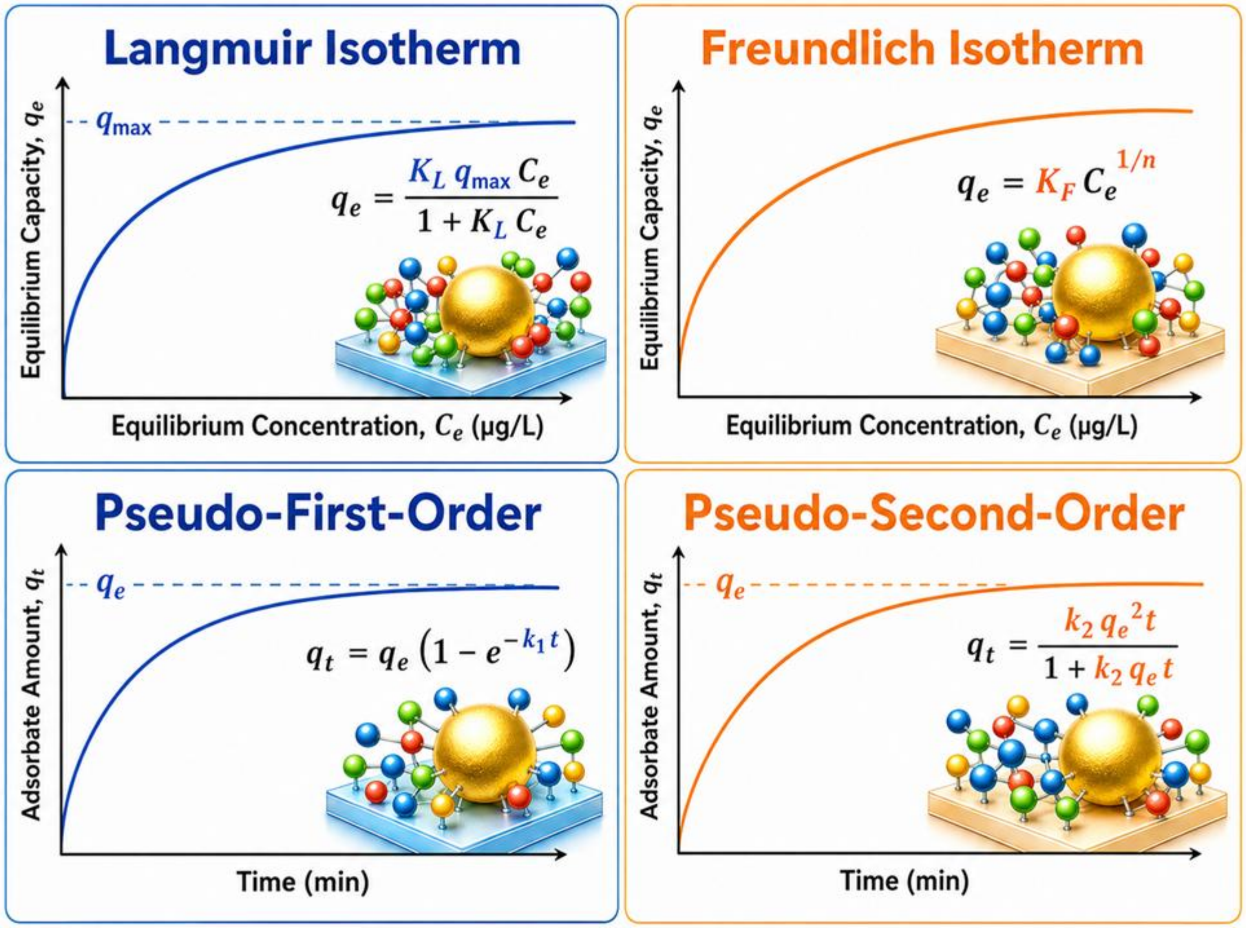

Figure S5. Adsorption isotherm and kinetic models for plasmonic sensing. Top: Langmuir and Freundlich isotherms (equilibrium, surface heterogeneity). Bottom: pseudo-first-order and pseudo-second-order kinetics (time-dependent adsorption). These models link adsorption capacity and rate to spectral shifts and intensity changes in plasmonic sensors.

Figure S6 presents field-deployable sampling strategies for water, soil, and air integrated with portable plasmonic sensors. Each pathway includes sample collection, pretreatment (filtration or extraction), and detection via adsorption-induced optical responses. The schematic emphasizes that analyte adsorption onto plasmonic substrates enables rapid, on-site environmental monitoring.

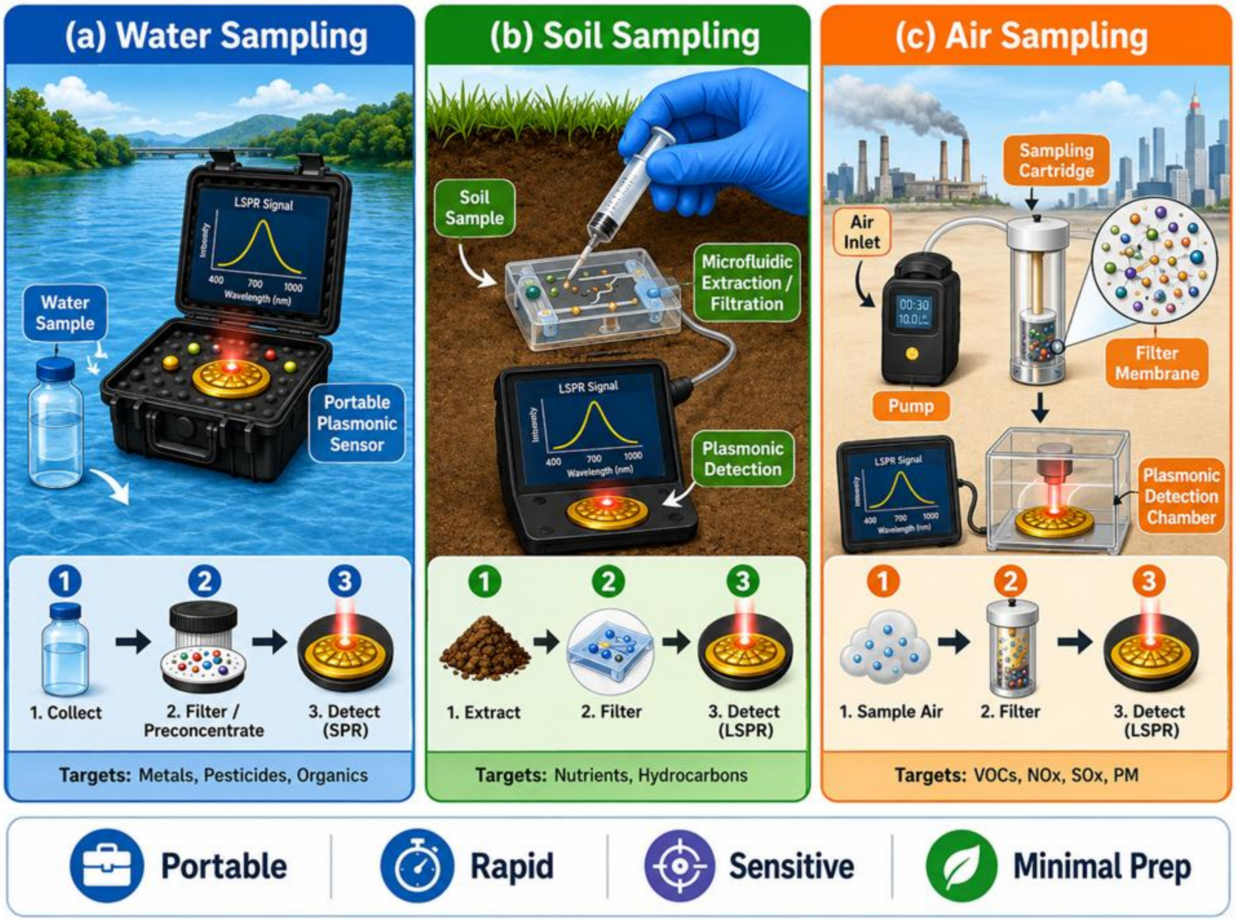

Figure S6. Field sampling strategies for plasmonic environmental sensing. (a) Water – filtration membranes with portable plasmonic devices. (b) Soil – microfluidic extraction integrated with plasmonic detection chambers. (c) Air – cartridges/filters for airborne species, followed by intensity/wavelength-based plasmonic detection. All pathways link sample collection and preconcentration to adsorption-driven optical readouts for rapid on-site analysis.

Figure S7 summarizes four major technological domains enabled by plasmonic adsorption and enhanced electromagnetic fields: ultrasensitive biosensing, plasmon-assisted photocatalysis and energy conversion, optical data storage and computing, and environmental remediation. These examples demonstrate that adsorption-controlled plasmonic interfaces can support applications across diagnostics, energy, information technology, and environmental protection.

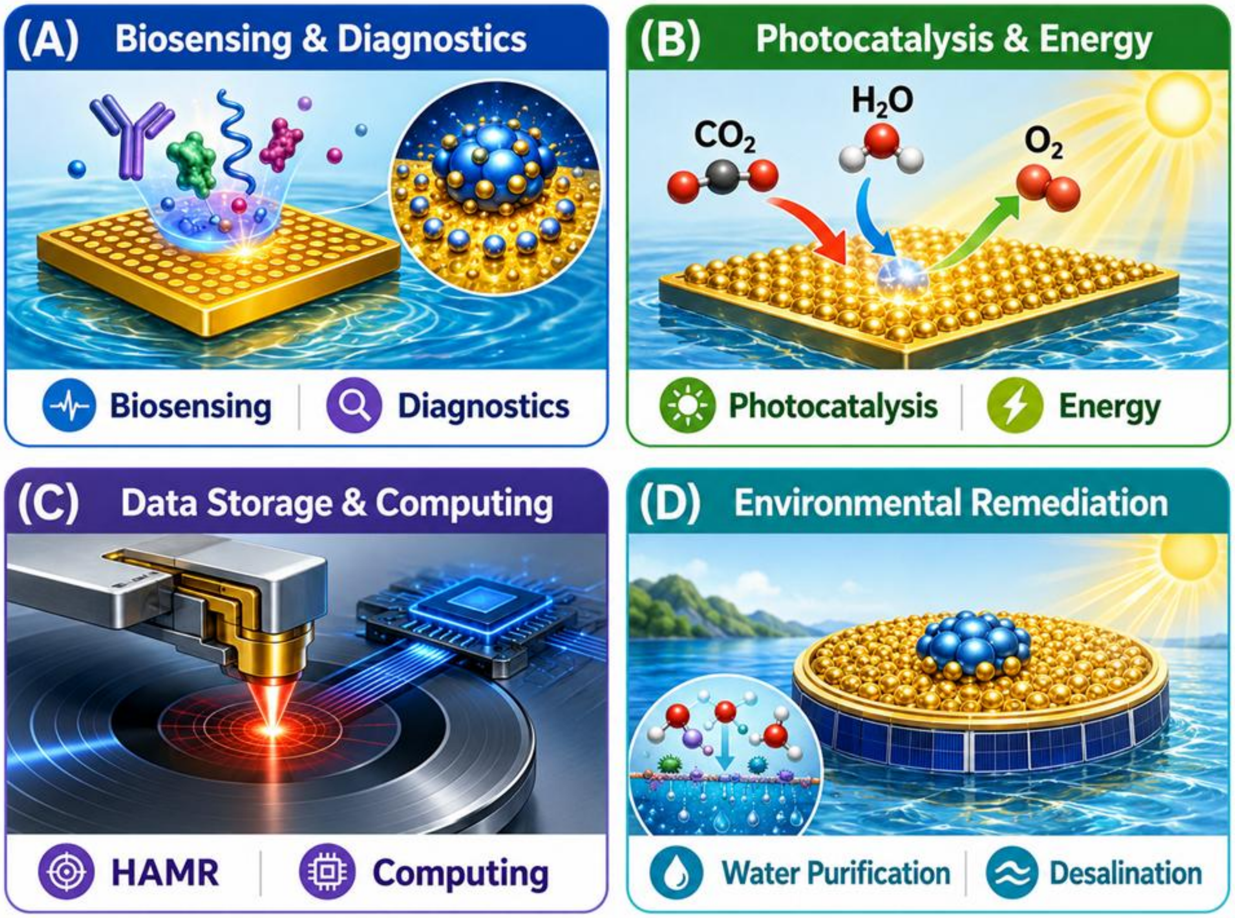

Figure S7. Plasmonic adsorption-enabled applications. (A) Biosensing – functionalized Au nanostructures capture biomolecules; adsorption-induced shifts or enhanced signals enable ultrasensitive detection. (B) Photocatalysis/energy – plasmon–semiconductor systems generate hot carriers for fuel production or pollutant degradation. (C) Data storage/computing – HAMR near-field transducer, plasmonic waveguides for on-chip communication. (D) Environmental remediation – solar-driven plasmonic absorbers for water purification and desalination.

Figure S8 highlights emerging plasmonic remediation technologies for the treatment of water, air, soil, and microplastics. Pollutants adsorb onto plasmonic nanostructures, where near-field enhancement, photothermal heating, and hot-carrier transfer drive degradation, sterilization, or purification. These light-powered and reagent-minimized strategies indicate a future in which plasmonic adsorption supports both environmental monitoring and sustainable cleanup.

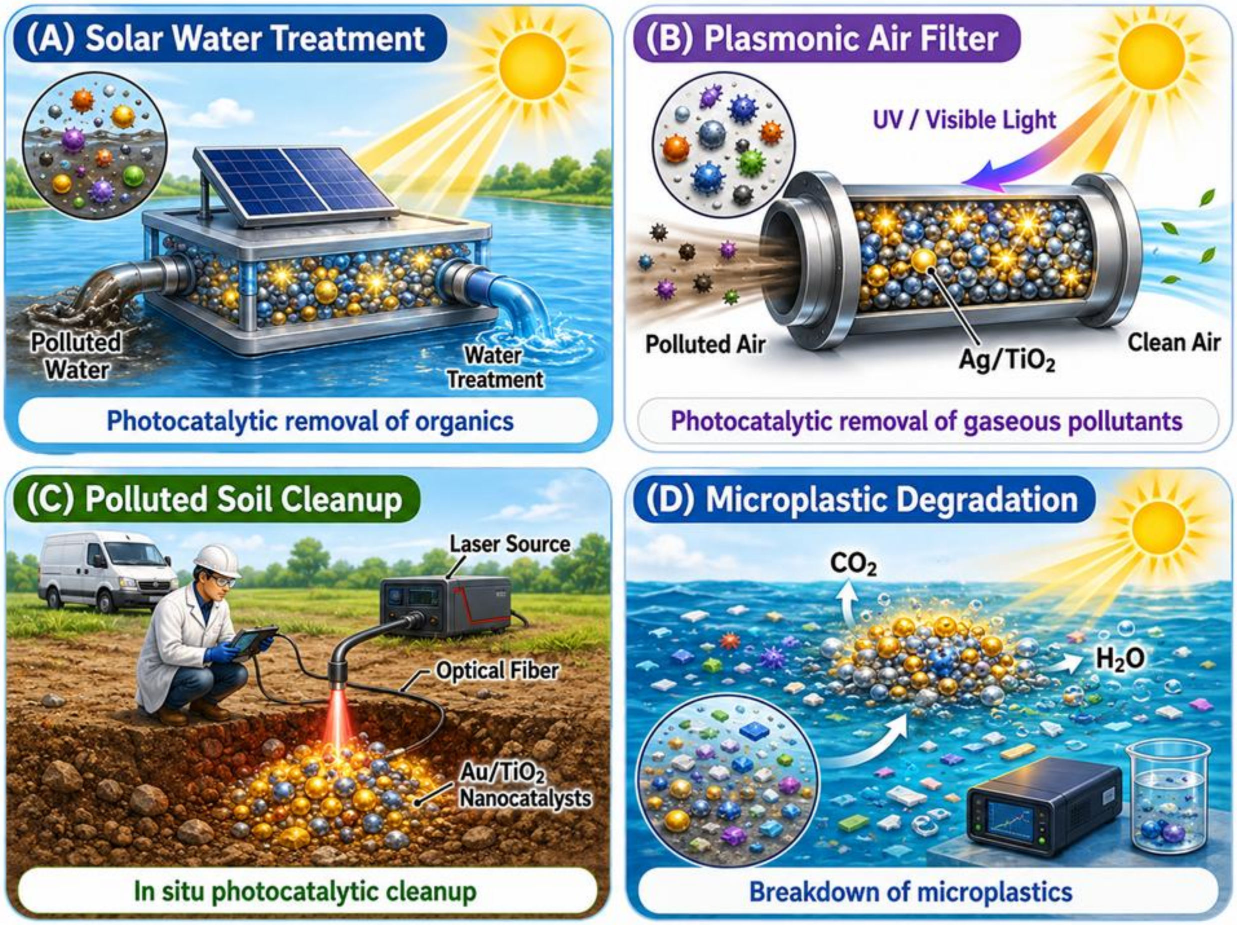

Figure S8. Plasmonic adsorption-enabled environmental remediation. (A) Solar water treatment – plasmonic absorbers generate heat/hot carriers for organic pollutant degradation. (B) Air filtration – nanoparticle-decorated filters adsorb and photocatalytically decompose VOCs/NO_2 under visible light. (C) Soil remediation – optical fiber-delivered light activates plasmonic photocatalysts to break down subsurface contaminants. (D) Microplastic degradation – plasmonic nanoreactors use local heating and hot-carrier chemistry to decompose polymer fragments under solar illumination.

Figure S9 summarizes this emerging synergy, showing how AI/ML can improve spectral analysis, noise suppression, nanostructure optimization, and autonomous decision-making in plasmonic adsorption-based sensing.

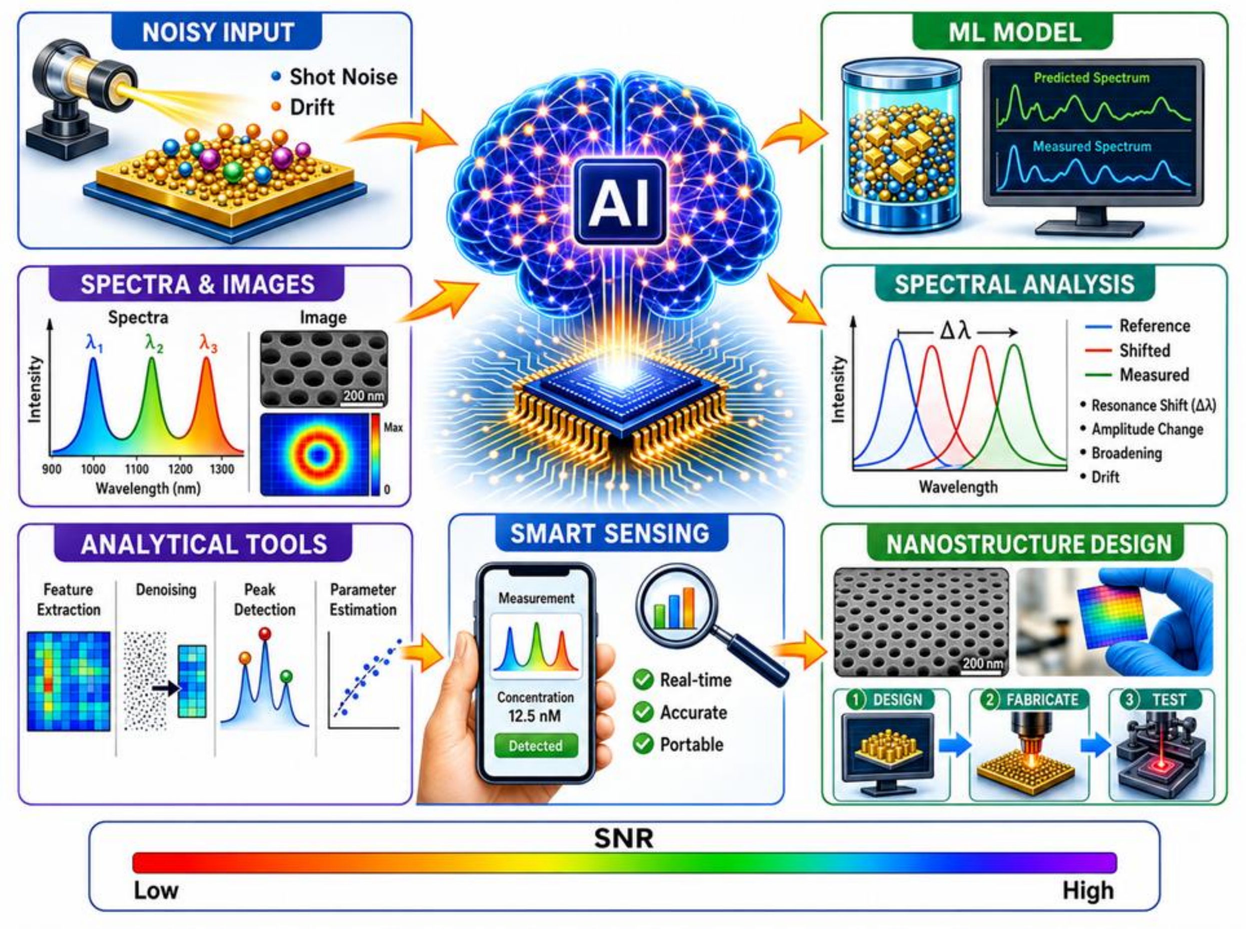

Figure S9. AI/ML integration with plasmonic sensing. Sensor data (resonance spectra, SERS fingerprints, images) are processed by ML algorithms to extract patterns, identify analytes, and suppress noise. Trained models enable automated classification, quantitative prediction, and real-time decision-making. AI feedback further optimizes nanostructure design and experimental parameters, forming a closed-loop intelligent sensing system.

Figure S10 outlines the commercialization roadmap for plasmonics-enhanced adsorption sensors, progressing from laboratory research to field deployment. Key stages include prototyping, scalable manufacturing, cost reduction, calibration and standardization, regulatory approval, user-centered design, and IoT/AI-enabled monitoring. The major conclusion is that commercialization requires parallel progress in science, engineering, manufacturing, data systems, and regulation. If these elements are integrated successfully, plasmonic adsorption technologies can evolve from high-performance research tools into practical platforms for environmental monitoring, diagnostics, food safety, industrial sensing, and smart infrastructure.

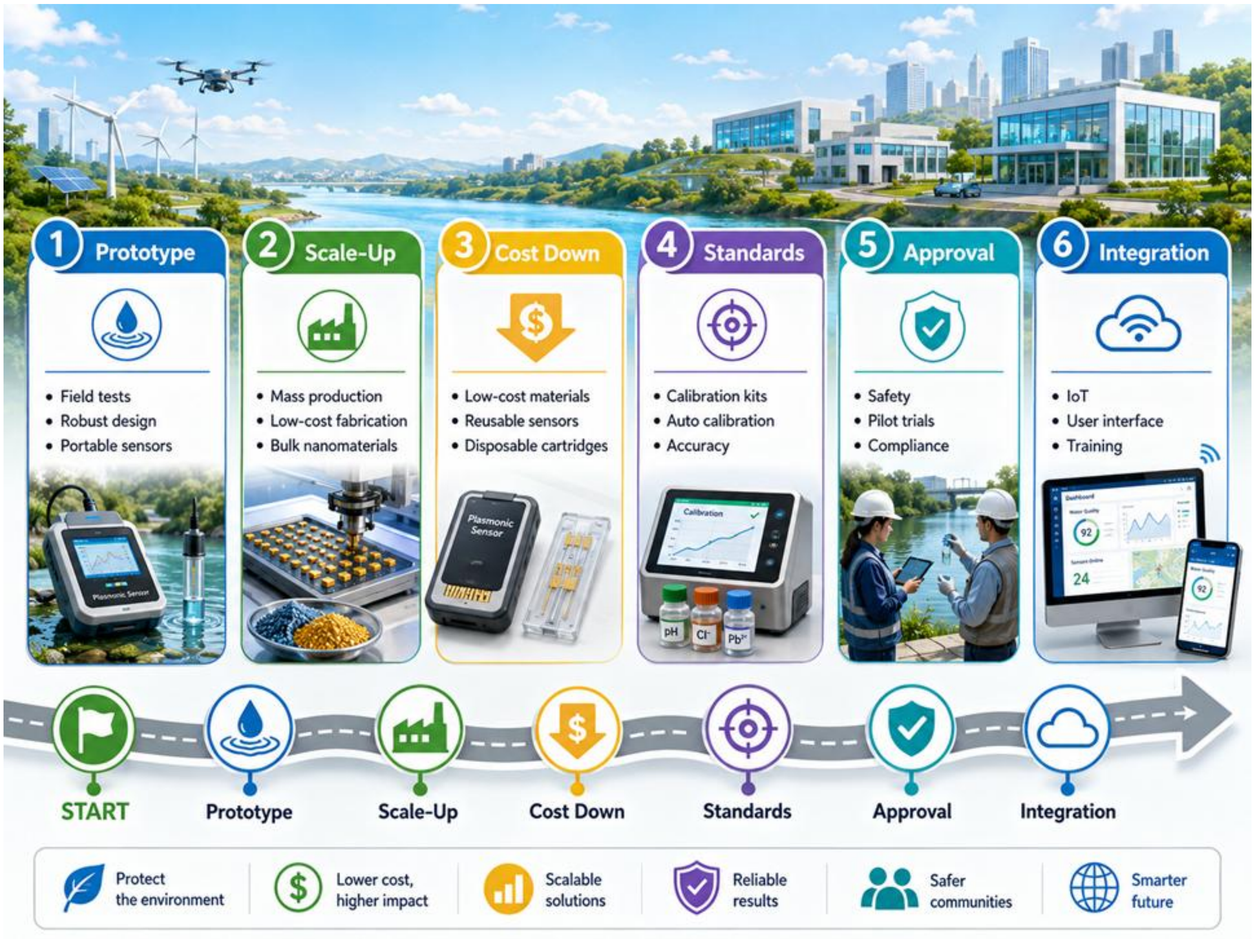

Figure S10. Commercialization roadmap for plasmonic adsorption sensors. Stages: (i) prototyping/validation; (ii) scalable manufacturing; (iii) cost reduction; (iv) calibration/standardization; (v) regulatory approval; (vi) user interface development. Integration with IoT and AI enables large-scale environmental deployment.

Reference

1. Chen, L., J. Li, and L. Chen, *Colorimetric Detection of Mercury Species Based on Functionalized Gold Nanoparticles.* ACS Applied Materials & Interfaces, 2014. **6**(18): p. 15897-15904.
2. Wu, B., et al., *Gold nanoparticle-enhanced D-shaped optical fiber sensor for mercury ion detection.* Analytical Methods, 2024. **16**(34): p. 5872-5882.
3. Gudagunti, F.D., et al. *Biosensor for the Characterization of Gene Expression in Cells*. Chemosensors, 2019. **7**, 60 DOI: 10.3390/chemosensors7040060.

4. Xu, X., et al., *Colorimetric detection of mercury ion (Hg2+) based on DNA oligonucleotides and unmodified gold nanoparticles sensing system with a tunable detection range.* Biosensors and Bioelectronics, 2009. **24**(10): p. 3153-3158.
5. Hu, W., et al., *Ultrastable liquid crystalline blue phase from molecular synergistic self-assembly.* Nat Commun, 2021. **12**(1): p. 1440.
6. Castro-Grijalba, A., et al., *SERS-Based Molecularly Imprinted Plasmonic Sensor for Highly Sensitive PAH Detection.* ACS Sensors, 2020. **5**(3): p. 693-702.
7. Lee, M., et al., *Subnanomolar Sensitivity of Filter Paper-Based SERS Sensor for Pesticide Detection by Hydrophobicity Change of Paper Surface.* ACS Sensors, 2018. **3**(1): p. 151-159.
8. Bhardwaj, K. and A. Jaiswal, *Plasmonic 3-D wrinkled polymeric shrink film-based SERS substrates for pesticide detection on real-world surfaces.* Analyst, 2023. **148**(3): p. 562-572.
9. Schiavi, S., et al. *Plasmonic Nanomaterials for Micro- and Nanoplastics Detection*. Applied Sciences, 2023. **13**, 9291 DOI: 10.3390/app13169291.
10. Rebelo, P., et al. *Molecularly Imprinted Plasmonic Sensors for the Determination of Environmental Water Contaminants: A Review*. Chemosensors, 2023. **11**, 318 DOI: 10.3390/chemosensors11060318.
11. He, C., et al. *Volatile Organic Compound Vapour Measurements Using a Localised Surface Plasmon Resonance Optical Fibre Sensor Decorated with a Metal-Organic Framework*. Sensors, 2021. **21**, 1420 DOI: 10.3390/s21041420.
12. Gaiardo, A., et al., *ZnO and Au/ZnO thin films: Room-temperature chemoresistive properties for gas sensing applications.* Sensors and Actuators B: Chemical, 2016. **237**.
13. Shen, Y., A. Tissot, and C. Serre, *Recent progress on MOF-based optical sensors for VOC sensing.* Chemical Science, 2022. **13**(47): p. 13978-14007.
14. Yang, P.-C., T. Wu, and Y.-W. Lin *Label-Free Colorimetric Detection of Mercury (II) Ions Based on Gold Nanocatalysis*. Sensors, 2018. **18**, 2807 DOI: 10.3390/s18092807.
